# Local synthesis of the phosphatidylinositol-3,4-bisphosphate lipid drives focal adhesion turnover

**DOI:** 10.1016/j.devcel.2022.06.011

**Published:** 2022-07-08

**Authors:** York Posor, Charis Kampyli, Benoit Bilanges, Sushila Ganguli, Philipp A. Koch, Alexander Wallroth, Daniele Morelli, Michalina Jenkins, Samira Alliouachene, Elitza Deltcheva, Buzz Baum, Volker Haucke, Bart Vanhaesebroeck

**Affiliations:** 1 UCL Cancer Institute, University College London, London WC1E 6DD, UK; 2 MRC Laboratory for Molecular Biology, University College London, London WC1E 6BS, UK; 3 Leibniz-Forschungsinstitut für Molekulare Pharmakologie (FMP), Berlin 13125, Germany; 4 MRC Laboratory of Molecular Cell Biology, University of Cambridge, Cambridge CB2 0QH, UK; 5 Faculty of Biology, Chemistry, and Pharmacy, Freie Universität Berlin, Berlin 14195, Germany

## Abstract

Focal adhesions are multifunctional organelles that couple cell-matrix adhesion to cytoskeletal force transmission and signaling and to steer cell migration and collective cell behavior. Whereas proteomic changes at focal adhesions are well understood, little is known about signaling lipids in focal adhesion dynamics. Through the characterization of cells from mice with a kinase-inactivating point mutation in the class II PI3K-C2β, we find that generation of the phosphatidylinositol-3,4-bisphosphate (PtdIns(3,4)P_2_) membrane lipid promotes focal adhesion disassembly in response to changing environmental conditions. We show that reduced growth factor signaling sensed by protein kinase N, an mTORC2 target and effector of RhoA, synergizes with the adhesion disassembly factor DEPDC1B to induce local synthesis of PtdIns(3,4)P_2_ by PI3K-C2β. PtdIns(3,4)P_2_ then promotes turnover of RhoA-dependent stress fibers by recruiting the PtdIns(3,4)P_2_-dependent RhoA-GTPase-activating protein ARAP3. Our findings uncover a pathway by which cessation of growth factor signaling facilitates cell-matrix adhesion disassembly via a phosphoinositide lipid switch.

## Graphical abstract

**Graphical abstract:**
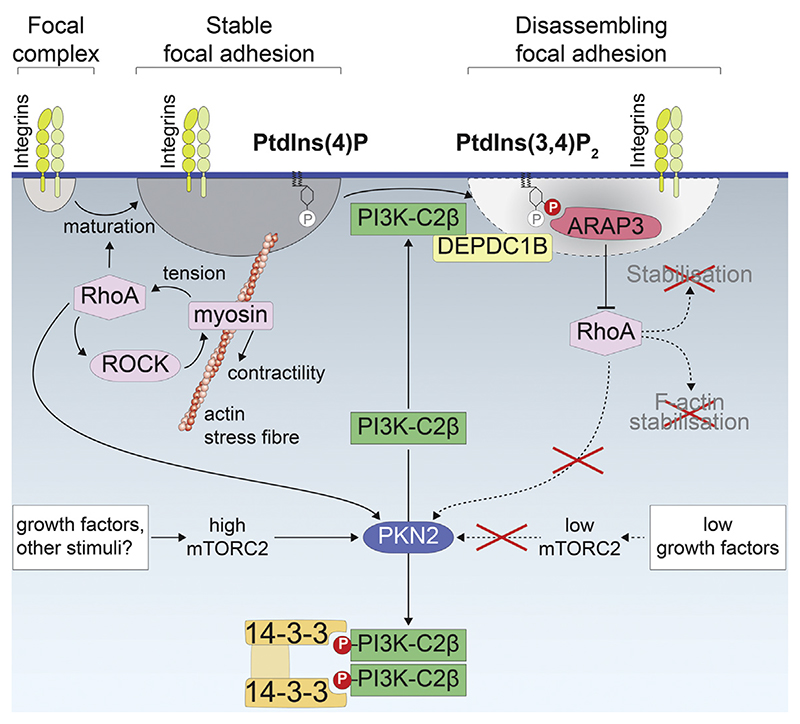

## Introduction

Changing cell shape during cell-cycle progression, cell migration, and in tissue and organ development requires cells to remodel their contacts with the extracellular matrix. These contacts termed focal adhesions (FAs) are dynamic integrin-dependent organelles that link the actin cytoskeleton to the extracellular matrix and transmit contractile forces to the substratum (Pandya et al., 2017; Parsons et al., 2010). The maturation of newly formed adhesions into larger and more stable FAs depends on attachment to F-actin fibers and on actomyosin contractility (Parsons et al., 2010; Revach et al., 2020). Stress fiber formation and contractility are positively regulated by the small GTPase RhoA in its active, GTP-bound state, and its effector Rho-associated coiled-coil-containing kinase (ROCK) (Ridley, 2015). In turn, guanine nucleotide exchange factors (GEFs) recruited to FAs in response to tension can perpetuate RhoA activation (Guilluy et al., 2011; Lawson and Ridley, 2018), leading to adhesion stabilization. However, for cells to move, a dynamic turnover of adhesions and balanced regulation of RhoA is required (Lawson and Ridley, 2018; Ridley, 2015). A large number of proteins have been implicated in FA disassembly (Kuo et al., 2011; Wehrle-Haller, 2012), including modulators of cytoskeletal tension and RhoA and Rac1 activities (Lawson and Burridge, 2014) and a range of other pathways (Ezratty et al., 2009; Franco et al., 2004; Kenific et al., 2016; Seetharaman and Etienne-Manneville, 2019; Sharifi et al., 2016; Stehbens et al., 2014). How the composition of the plasma membrane changes during adhesion turnover is however largely unknown.

Phosphoinositide lipids are key regulators of the actin cytoskeleton and membrane dynamics driven by filamentous F-actin (Balla, 2013; Posor et al., 2022; Tsujita and Itoh, 2015). A key lipid in this context is phosphatidylinositol-(4,5)-bisphosphate (PtdIns(4,5)P_2_), produced by the PtdIns(4)P-5 kinase PIPKIγ at FAs (Di Paolo et al., 2022). PtdIns(4,5)P_2_ binds to and activates key adhesion components such as talin and vinculin (Di Paolo et al., 2002; Legate et al., 2011), thereby promoting adhesion formation and anchoring of actin fibers (Senju and Lappalainen, 2019). In contrast, the roles of phosphoinositides in adhesion turn-over are poorly understood. Disassembly of adhesions may in part rely on PtdIns(4,5)P_2_ as a cofactor to recruit the calpain protease (Franco et al., 2004; Shao et al., 2006) and to stimulate clathrin-dependent endocytosis of integrins, signaling cell-matrix adhesion receptors (Chao et al., 2010; Ezratty et al., 2009).

Phosphoinositides are known to be employed in a switch-like manner, where fate decisions of cellular structures or organelles are governed by a change in local phosphoinositide synthesis (Posor et al., 2022). This is exemplified by a switch from PtdIns(4,5)P_2_ to phosphatidylinositol-3,4-bisphosphate (PtdIns (3,4)P_2_) synthesis during clathrin-mediated endocytosis (Posor et al., 2013; Wang et al., 2019), or by cargo sorting at endosomes where a conversion of PtdIns(3)P to PtdIns(4)P promotes recycling (Ketel et al., 2016), whereas sorting along the degradative route is accompanied by consecutive formation of PtdIns(3)P and PtdIns(3,5)P_2_ (Schink et al., 2016). The established role for PtdIns(4,5)P_2_ formation in FA formation and stabilization would predict the existence of a switch to the formation of an alternate phosphoinositide lipid species during disassembly.

Possible candidates for a lipid switch triggering disassembly are phosphoinositide 3-kinases (PI3Ks), i.e., enzymes that synthesize 3-phosphoinositide lipids that dominate the endocytic and endolysosomal system. Among the PI3Ks, the class II PI3Ks have emerged as regulators of membrane dynamics at the interface of signaling and trafficking. In contrast to class I PI3K-driven PtdIns(3,4,5)P_3_/Akt/mTORC1 signaling, class II PI3Ks produce PtdIns(3)P and PtdIns(3,4)P_2_, most likely at specific subcellular locations (Bilanges et al., 2019; Gulluni et al., 2019). These include perinuclear lysosomes where PtdIns(3,4) P_2_ production by the class II PI3K-C2β isoform under conditions of mitogen starvation represses mTORC1 activity (Marat et al., 2017). This inhibition can be relieved by growth factor stimulation through phosphorylation of PI3K-C2β on T279 by protein kinase N2 (PKN2), which itself is activated by growth-factor-activated mTORC2 (Liu and Sabatini, 2020; Wallroth et al., 2019).

Here, we identify PtdIns(3,4)P_2_ formation by PI3K-C2β to promote adhesion disassembly and delineate both upstream and downstream pathway components, revealing a mechanism for how cessation of growth factor signaling regulates cell adhesion turnover through a phosphoinositide lipid switch.

## Results

### PI3K-C2β regulates focal adhesion disassembly

**Figure 1:**
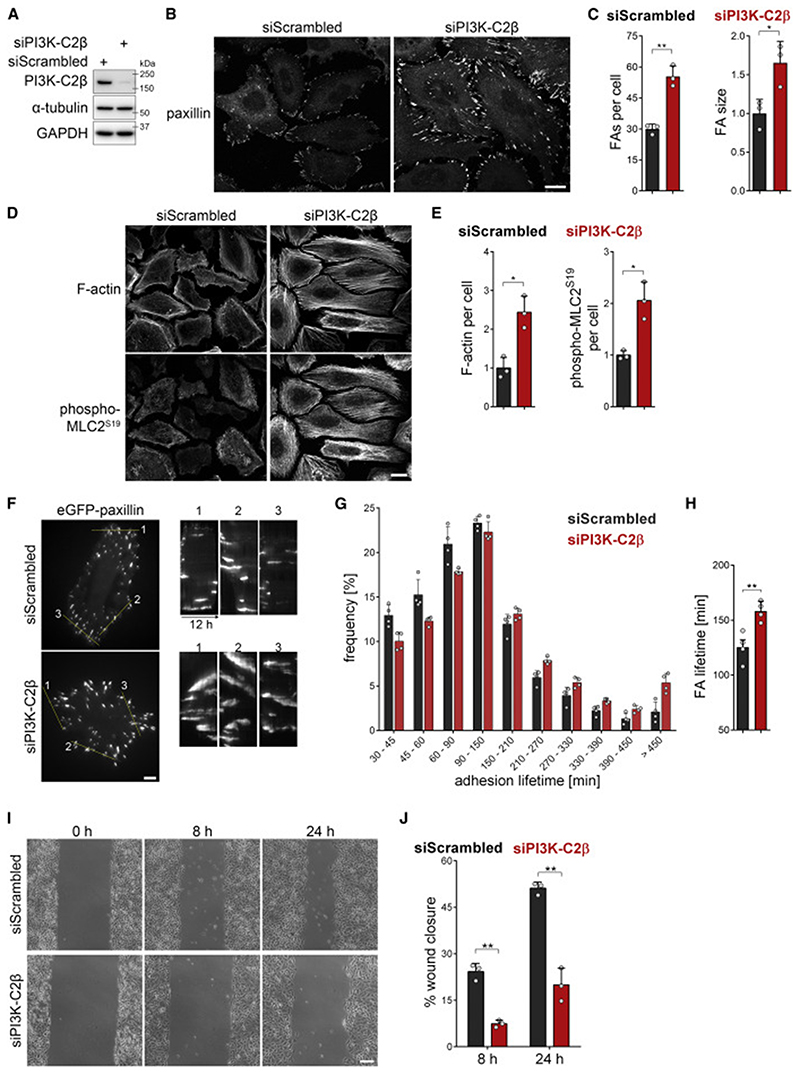
Accumulation of cell-matrix adhesions and dampened cell migration upon depletion of PI3K-C2β (A–E) Altered morphology of cell-matrix adhesions and actin stress fibers upon depletion of PI3K-C2β. PI3K-C2β-depleted HeLa cells were (B and C) stained for paxillin or (D and E) stained for MLC2 phospho-Ser^19^ and for F-actin using phalloidin (laser scanning confocal micro-scopy). (A) Immunoblots of cell extracts representative of at least 5 independent experiments. α-tubulin and GAPDH were detected as loading controls. (B and D) Scale bars, 20 μm. (E) Total fluorescence intensity per cell for phalloidin staining (F-actin) or MLC2 phospho-Ser^19^. (C and E) Mean + SD from n = 3 independent experiments, unpaired two-tailed t test with Welch’s correction. (F–H) Attenuated dynamics of cell-matrix adhesions in PI3K-C2β-depleted cells. PI3K-C2β-depleted HeLa cells transiently expressing eGFP-paxillin were imaged by TIRF microscopy at 5 min/frame for 12 h. (F) Representative kymographs along the lines drawn in the images on the left. Scale bars, 10 μm. (G) Frequency of lifetimes of paxillin-labeled structures present for at least 30 min. Data shown are means + SD from n = 4 independent experiments (total of 91 cells for siScrambled, 89 cells for siPI3K-C2β). (H) Average lifetime of paxillin-labeled adhesions analyzed as in (F). Mean + SEM from n = 4 independent experiments, unpaired two-tailed t test with Welch’s correction. (I and J) Impaired migration in PI3K-C2β-depleted HeLa cells. Scratch wound assay with wound closure assessed after 8 and 24 h in complete medium containing serum and mitomycin C to prevent cell proliferation. (I) Bar, 200 μm. (J) Mean + SD from n = 3 independent experiments, unpaired two-tailed t test with Welch’s correction. See also Figure S1. FA, focal adhesion.

When characterizing mouse embryonic fibroblasts (MEFs) derived from our previously generated mice bearing a kinase-in-activating point mutation in PI3K-C2β (Alliouachene et al., 2015), we observed an altered cell shape. PI3K-C2β kinase-dead (KD) mutant MEFs adopted a more spread morphology, appearing larger when adhering to the culture dish. We therefore analyzed the cell-matrix adhesions of these cells by staining for the adhesion component paxillin, which revealed an accumulation of FAs in PI3K-C2β-mutant MEFs (Figures S1A and S1B). To expand on this finding, we turned to an easily amenable model system and assessed the morphology of cell-matrix adhesions and the actin cytoskeleton in HeLa cells. PI3K-C2β-depleted HeLa cells cultured in serum-containing media displayed a striking accumulation of FAs, with both an increase in the number of adhesions per cell and an increase in the size of the individual adhesions (Figures 1A–1C, S1E, and S1F). This was accompanied by exacerbated formation of stress fibers and increased levels of Ser19-phosphorylated myosin light chain 2 (pMLC2^S19^), an indicator of actomyosin contractility (Figures 1D, 1E, and S1F). We made similar observations in the migratory breast cancer cell line MDA-MB-231 (Figures S1H–S1J), suggesting a conserved role for PI3K-C2β in the regulation of cell-matrix adhesions across different cell types.

To investigate whether these morphological changes correlate with altered turnover of cell-matrix adhesions, we analyzed the dynamics of the localization of eGFP-paxillin, an established marker of FAs, in live cells. Depletion of PI3K-C2β potently reduced the rate of adhesion turnover, as illustrated by a pronounced shift in the distribution and average lifetimes of FAs (Figures 1F–1H). These observations suggest that PI3K-C2β is required for homeostatic regulation and dynamic remodeling of cell-matrix adhesions. To test the functional impact of such altered cell-matrix adhesion, we assessed the ability of cells to migrate in a scratch wound assay. PI3K-C2β depletion led to a strongly delayed wound closure, indicating impaired cell motility (Figures 1l, 1J, and S1G), in agreement with earlier observations (Domin et al., 2005; Katso et al., 2006; Maffucci et al., 2005). We made similar observations in PI3K-C2β KD mutant MEFs (Figures S1C and S1D).

**Figure 2:**
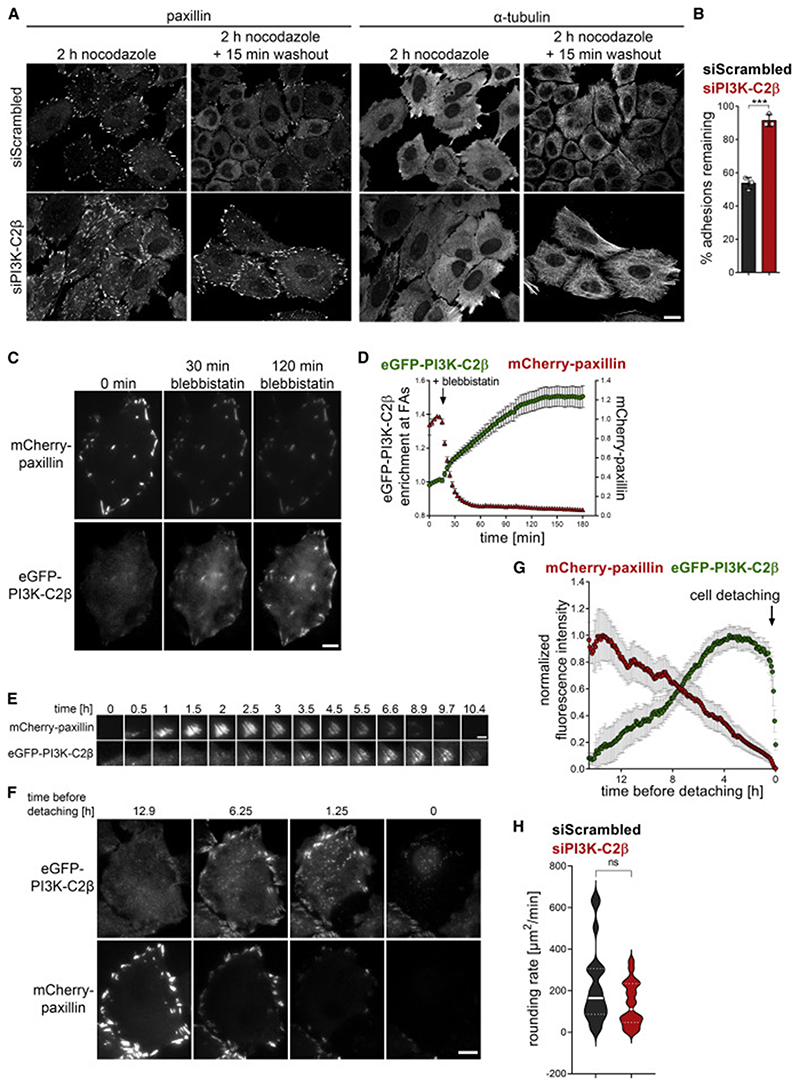
PI3K-C2β is required locally for cell-matrix adhesion disassembly (A and B) Impaired cell-matrix adhesion disassembly in PI3K-C2β-depleted HeLa cells. Adhesion disassembly was stalled by nocodazole treatment. Synchronous adhesion disassembly was then triggered by nocodazole washout for 15 min. Cells stained for paxillin and α-tubulin were imaged by spinning disk confocal microscopy. (A) Scale bars, 20 μm. (B) Adhesions remaining 15 min after nocodazole washout (in percent of adhesions at 2 h nocodazole treatment); mean + SD from n = 3 independent experiments, unpaired two-tailed t test with Welch’s correction. (C and D) PI3K-C2β is recruited to disassembling cell-matrix adhesions. HeLa cells with endogenously tagged eGFP-PI3K-C2β transiently expressing mCherry-paxillin were imaged by TIRF microscopy at 3 min/frame. After five frames, cells were treated with 25-μM blebbistatin to trigger adhesion disassembly and imaged for a total of 3 h. (C) Scale bars, 10 μm. (D) Enrichment of eGFP-PI3K-C2β (mean intensity at adhesions over mean intensity in rest of the cell) and mCherry-paxillin intensity at adhesions as labeled by paxillin at baseline. Mean ± 95% confidence interval from n = 75 cells from three independent experiments. (E–G) eGFP-PI3K-C2β is recruited to spontaneously disassembling cell-matrix adhesions. Cells as in (C) were imaged by TIRF microscopy for 16 h at 5 min/frame. (E) Example of a single focal adhesion forming and disassembling. Scale bars, 3 μm. (F) Example of a cell preparing to detach for cytokinesis. Scale bars, 10 μm. (G) Quantification of eGFP-PI3K-C2β recruitment to adhesions in cells detaching for cytokinesis as in (F) (signal from all, not individual adhesions). mCherry-paxillin and eGFP-PI3K-C2β fluorescence was quantified at adhesions as defined by a maximum-intensity projection of the mCherry-paxillin signal across all time points. Intensity traces were aligned by the time point of detachment. Mean ± 95% confidence interval from n = 63 cells from three independent experiments. (H) Mitotic rounding rate is not significantly affected in HeLa cells depleted of PI3K-C2β. Cells were imaged by phase-contrast time-lapse microscopy and the rate of cell area change at the time of nuclear envelope breakdown was measured. Violin plots representing distribution of data points from n = 20 cells per condition; thick lines indicate median, thin dashed lines indicate first and third quartiles. See also Figure S2. FAs, focal adhesions.

Cell-matrix adhesion dynamics are critically controlled by the rates of *de novo* formation of adhesions and the disassembly of existing adhesions. To understand which of these two basic components of cell-matrix adhesion turnover is affected upon PI3K-C2β depletion, we assessed these processes separately. To address *de novo* adhesion formation, we trypsinized cells and analyzed the number of adhesions per cell at different time points after re-seeding. Depletion of PI3K-C2β did not alter the number of adhesions or cell area at early time points after reseeding (30 min or 1 h) (Figures S2A–S2C). An increased accumulation of FAs as well as an increase in cell area were observed 6 h post re-seeding of PI3K-C2β-depleted cells compared with control cells (Figures S2A–S2C). This suggests that the *de novo* formation of adhesions proceeds unperturbed in the absence of PI3K-C2β, with differences only emerging over time as adhesions turn over. We therefore directly probed FA disassembly using the well-established nocodazole-washout protocol, which is based on the notion that an intact microtubule network is required for FA disassembly (Ezratty et al., 2005; Feutlinske et al., 2015). Microtubule depolymerization by nocodazole treatment inhibits FA disassembly, with washout of this compound triggering synchronized FA disassembly. After 15 min of nocodazole washout, PI3K-C2β-depleted cells retained more than 90% of their adhesions, while ~50% of the adhesions in control cells had undergone turnover (Figures 2A and 2B). A comparable impairment of FA disassembly was observed in MEFs (Figures S2D and S2E). These data show that the increased number and size of adhesions upon PI3K-C2β down-regulation is due to the selective impairment of FA disassembly.

We next asked whether PI3K-C2β plays a local role at cell-matrix adhesions to facilitate their disassembly. For this purpose, we turned to genome-edited HeLa cells that express eGFP-tagged endogenous PI3K-C2β (Wallroth et al., 2019). Of note, endogenous tagging of PI3K-C2β was only successful in HeLa cells of the Kyoto subclone, which display slower adhesion turnover and less migratory capacity than HeLa cells (American type culture collection #CCL-2). Whereas this limits the possible scope of experiments with these cells, it allowed us to investigate the subcellular localization of the endogenous PI3K-C2β enzyme. In these cells, PI3K-C2β displayed only sporadic co-localization with adhesions labeled with paxillin viewed by total internal reflection fluorescence (TIRF) microscopy in cells kept under steady-state culture conditions (Figure 2C, left panel). In light of our findings above, we reasoned that PI3K-C2β might selectively localize to disassembling adhesions. To capture this step of the adhesion process, we induced synchronous adhesion disassembly by treating cells with the myosin II inhibitor blebbistatin, an established procedure to relax actin stress fibers and to trigger the dismantling of mature FAs (Feutlinske et al., 2015; Parsons et al., 2010). As expected, loss of contractility upon blebbistatin addition triggered the rapid disappearance of paxillin from adhesions, correlating with a striking accumulation of eGFP-PI3K-C2β at these sites (Figures 2C and 2D; Video S1; note that the regions of interest designating adhesions were defined in the first frame and kept constant, i.e., newly forming adhesions were not considered). Recruitment of eGFP-PI3K-C2β to adhesions was also observed during spontaneous physiological adhesion turnover in cells imaged over extended periods of time (Figure 2E). Interestingly, PI3K-C2β was absent from FAs during adhesion nucleation and growth, while the onset of PI3K-C2β accumulation strikingly coincided with the decline of the paxillin signal (Figure 2E). PI3K-C2β then remained present until after paxillin had entirely disappeared from the adhesion (Figure 2E). To quantify the recruitment during physiological adhesion disassembly, we took advantage of synchronized disassembly in cells preparing to go through cell division. The continuous decline in paxillin signal at cell adhesions before detachment correlated with a steady accumulation of eGFP-PI3K-C2β, which plateaued well before cells detached (Figures 2F and 2G; Video S2), suggesting a role of PI3K-C2β in the lead-up to mitotic rounding. This is consistent with only a minor tendency to a slowed rate of cell rounding upon depletion of PI3K-C2β as measured by the rate of cell area change at the time of nuclear envelope breakdown (Figure 2H). Collectively, these data show that PI3K-C2β is specifically recruited to disassembling adhesions, in response to cell-intrinsic (i.e., before cell division) or -extrinsic cues (see below).

### Recruitment of PI3K-C2β to adhesions is mediated by the adhesion disassembly factor DEPDC1B and regulated by protein kinase N downstream of mTORC2

**Figure 3:**
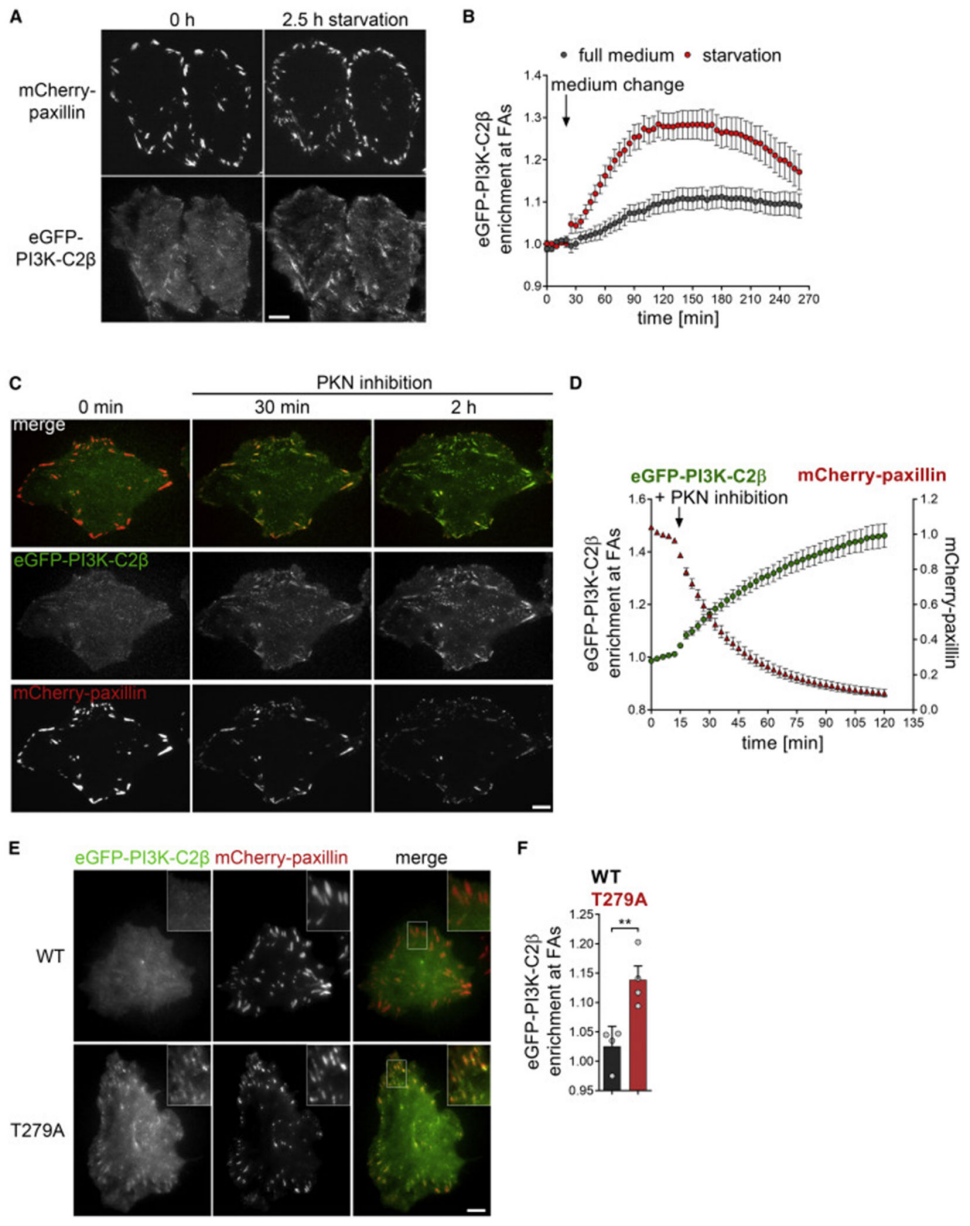
PI3K-C2β recruitment to adhesions is regulated by PKN (A and B) Starvation induces recruitment of PI3K-C2β to cell-matrix adhesions. HeLa cells with endogenous eGFP-PI3K-C2β transiently expressing mCherry-paxillin were imaged by TIRF microscopy at 5 min/frame. After 5 frames, medium was replaced with fresh complete serum-containing culture medium or starvation medium (EBSS with glucose, no serum or amino acids). (A) Scale bars, 10 μm. (B) Enrichment of eGFP-PI3K-C2β (mean intensity at adhesions over mean intensity in rest of the cell) at adhesions as labeled by paxillin in frame 1. Mean ± 95% confidence interval from n = 82 cells (complete medium) or n = 85 cells (starvation) from three independent experiments. (C and D) Inhibition of PKN triggers accumulation of PI3K-C2β at cell-matrix adhesions. HeLa cells as in (A) cultured in complete medium containing serum were imaged by TIRF microscopy at 3 min/frame. After 5 frames, PKN was inhibited by addition of 1 μM PKC412 + 2 μM Cdk1/2 inhibitor III. (C) Scale bars, 10 μm. (D) Enrichment of eGFP-PI3K-C2β as in (A). Mean ± 95% confidence interval from n = 84 positions (multiple cells per position) from three independent experiments. (E and F) The T279A mutant of PI3K-C2β, which cannot be phosphorylated by PKN2, displays increased basal localization at cell-matrix adhesions. Live HeLa cells transiently expressing mCherry-paxillin and either WT or T279AeGFP-PI3K-C2β were imaged by TIRF microscopy. (E) Bar, 10 μm. (F) Enrichment of eGFP-PI3K-C2β at mCherry-paxillin-labeled adhesions (as in C). Mean + SEM from n = 4 independent experiments, unpaired two-tailed t test with Welch’s correction. See also Figure S4. FAs, focal adhesions.

PI3K-C2β has been implicated in the cellular response to changing environmental conditions sensed by mTORC2 (Wallroth et al., 2019), including signaling from growth factor receptors that leads to mTORC2 activation. We therefore asked whether the function of PI3K-C2β at cell-matrix adhesions is regulated in response to nutrient starvation by monitoring endogenous eGFP-PI3K-C2β in genome-engineered HeLa cells cultured in EBSS (Earle’s balanced salt solution, contains glucose but no serum or amino acids). Indeed, such starvation triggered a sustained accumulation of eGFP-PI3K-C2β at cell-matrix adhesions, reaching a plateau at about 90 min after the onset of starvation (Figures 3A and 3B).

Recent work has established that the recruitment of PI3K-C2β to membranes is inhibited by PKN2, which is activated downstream of mTORC2 signaling and RhoA activation (Wallroth et al., 2019). This inhibitory mechanism involves phosphorylation of PI3K-C2β on T279 by PKN2, which leads to inactivation of PI3K-C2β through sequestration by 14-3-3 proteins (Wallroth et al., 2019). We used pharmacological inhibition of PKN2 (Wallroth et al., 2019) to test whether this protein kinase controls PI3K-C2β recruitment to cell-matrix adhesions. Pharmacological blockade of PKN2 caused the rapid and sustained recruitment of endogenous eGFP-PI3K-C2β to FAs (Figures 3C and 3D; Video S3). To address the role of PI3K-C2β phosphorylation at T279 by PKN2 by an independent approach, we compared the sub-cellular localization of transiently expressed eGFP-PI3K-C2β-wild-type (WT) or a PI3K-C2β-T279A mutant that cannot be phosphorylated by PKN (Wallroth et al., 2019). As shown in Figures 3E and 3F, eGFP-PI3K-C2β-T279A displayed increased enrichment at adhesions (labeled by mCherry-paxillin) in complete medium, further supporting a role of PKN2 in the recruitment of PI3K-C2β to FAs. Interestingly, PKN-inhibition-induced recruitment of PI3K-C2β to adhesions was mirrored by a sharp decline of the paxillin signal (Figures 3C and 3D), suggesting that loss of PKN activity is an important regulatory signal for induction of FA disassembly. Taken together, these observations suggest a model whereby PI3K-C2β is recruited to cell-matrix adhesions to promote adhesion turnover in response to environmental conditions that dampen mTORC2 activity.

**Figure 4:**
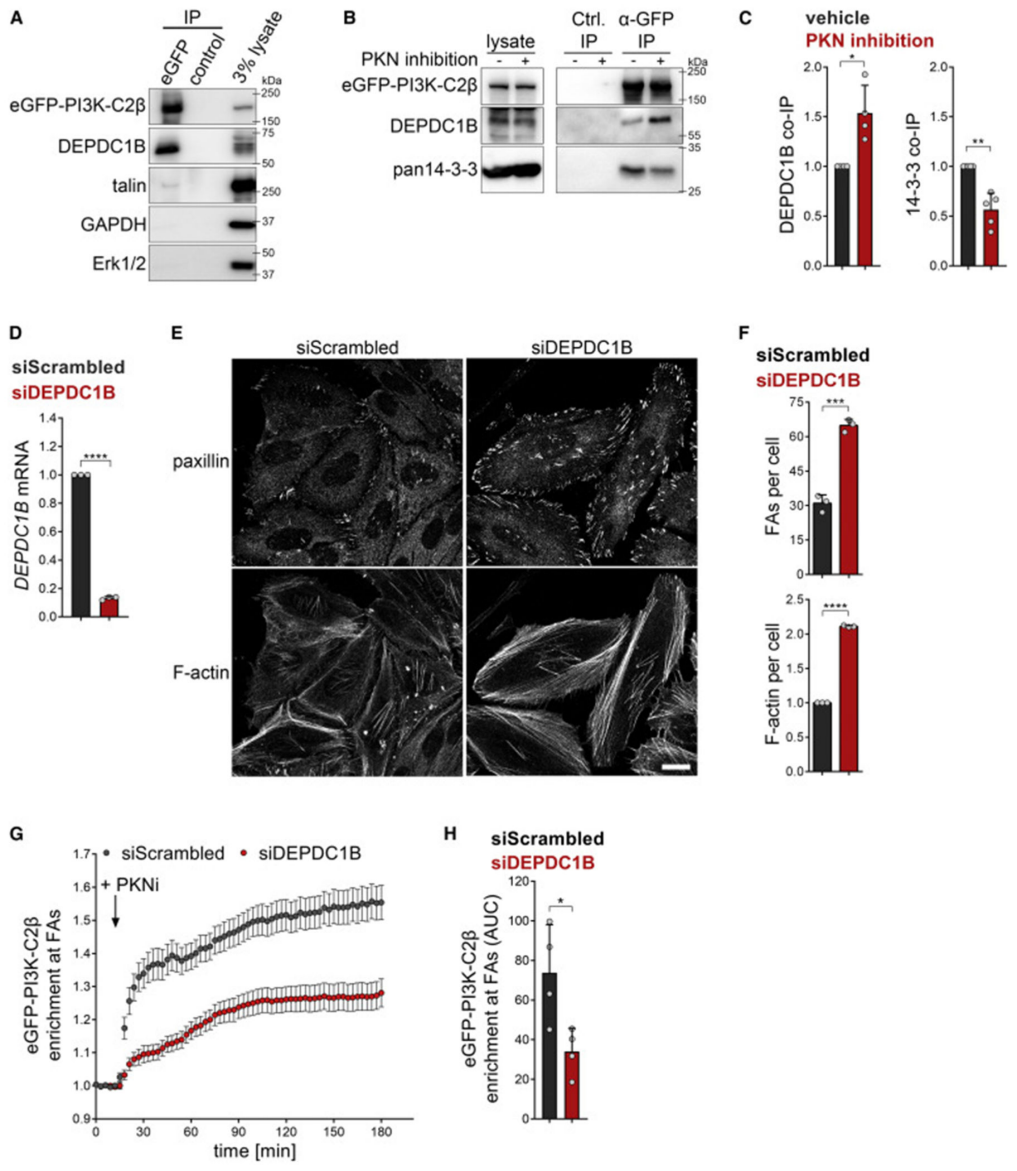
DEPDC1B recruits PI3K-C2β to disassembling cell-matrix adhesions (A) Endogenous PI3K-C2β and DEPDC1B form a complex in cells. Endogenous eGFP-PI3K-C2β was immunoprecipitated from HEK293 cells and strongly co-precipitated endogenous DEPDC1B, whereas talin co-purified weakly. GAPDH and Erk1/2 served as negative controls (data representing four independent experiments). (B and C) Upon inhibition of PKN, PI3K-C2β displays enhanced complex formation with DEPDC1B, whereas interaction with 14-3-3 proteins is decreased. Cells as in (A) were treated for 2 h with vehicle or PKN inhibitors (1-μM PKC412 + 2-μM Cdk1/2 inhibitor III) and subjected to immunoprecipitation using GFP-trap resin. (C) Densitometric quantification of immunoblots as shown in (B). Mean + SD from n = 4 (DEPDC1B) and n = 5 (14-3-3) independent experiments; one sample, two-tailed t test with hypothetical mean of 1.0. (D–F) Depletion of DEPDC1B using siRNAs phenocopies loss of PI3K-C2β. (D) DEPDC1B mRNA levels were determined by qRT-PCR; one sample, two-tailed t test with hypothetical mean of 1.0. (E) Cells were stained for paxillin and F-actin and imaged by confocal microscopy. Scale bars, 20 μm. (F) Mean + SD from n = 3 independent experiments, unpaired two-tailed t test with Welch’s correction (FAs per cell) or one sample, two-tailed t test with hypothetical mean of 1.0 (F-actin per cell). (G and H) Depletion of DEPDC1B using siRNAs attenuates recruitment of eGFP-PI3K-C2β to focal adhesions. HeLa cells with endogenous eGFP-PI3K-C2β transiently expressing mCherry-paxillin were imaged by TIRF microscopy at 3 min/frame. After 5 frames, PKN was inhibited as in (B). (G) Enrichment of eGFP-PI3K-C2β (mean intensity at adhesions over mean intensity in rest of the cell) at adhesions as labeled by paxillin in frame 1. Mean ± 95% confidence interval from n = 146 cells (scrambled siRNA) or n = 139 cells (siDEPDC1B). (H) Area under the curve (AUC) from data shown in (G). Mean + SD from n = 4 independent experiments, unpaired two-tailed t test with Welch’s correction. See also Figure S4. FAs, focal adhesions.

No molecular link between the adhesion-interactome and PI3K-C2β has been uncovered yet, raising the critical question of how PI3K-C2β is recruited to cell-matrix adhesions. To tackle this problem, we conducted a yeast-2-hybrid screen using the N terminus of PI3K-C2β as a bait. We identified DEPDC1B, a poorly characterized protein containing a DEP- (Dishevelled, Egl-10 and pleckstrin) domain and an inactive RhoGAP-like domain (Figure S3A). DEPDC1B associates with the transmembrane scaffold protein and tyrosine phosphatase PTPRF (protein tyrosine phosphatase receptor type F) (Marchesi et al., 2014), which localizes to FAs and organizes events related to adhesion and migration (Sarhan et al., 2016; Serra-Pagès et al., 1995). DEPDC1B has previously been implicated in facilitating FA disassembly upon cell detachment before mitosis (Marchesi et al., 2014). We first validated DEPDC1B as an interaction partner of PI3K-C2β using affinity-purification experiments. When co-expressed in HEK293T cells, 6×myc-PI3K-C2β co-immunoprecipitated with eGFP-DEPDC1B (Figure S3B). Conversely, immunoprecipitated eGFP-DEPDC1B co-purified with 6×myc-PI3K-C2β (Figure S3B). To confirm these findings under physiological conditions, we used genome-engineered HEK293T cells expressing endogenous eGFP-PI3K-C2β (Marat et al., 2017). Immunoprecipitation of eGFP-PI3K-C2β from these cells robustly co-purified endogenous DEPDC1B (Figure 4A). eGFP-PI3K-C2β immunoprecipitates also contained low levels of the adhesion scaffolding protein talin (Figure 4A). Enhancing FA recruitment of PI3K-C2β by inhibition of PKNs caused increased association of PI3K-C2β with DEPDC1B and a concomitantly reduced association with 14-3-3 proteins (Figures 4B and 4C). Taken together, these results show that PI3K-C2β and DEPDC1B form a complex in cells and that complex formation is enhanced under conditions of increased association of PI3K-C2β with FAs.

We next asked whether PI3K-C2β and DEPDC1B functionally co-operate to facilitate FA disassembly. Interestingly, depletion of DEPDC1B phenocopied loss of PI3K-C2β, with cells showing an accumulation of FAs and a more pronounced formation of actin stress fibers (Figures 4D–4F). Similar observations were made in cells in which the DEPDC1B gene had been disrupted using clustered regularly interspaced short palindromic repeats (CRISPRs)-Cas9 editing (Figure S4A). Re-expression of eGFP-DEPDC1B in two separate knockout clones reduced the number of FAs and actin stress fiber formation to the levels seen in WT cells (Figures S4B and S4C). To test whether DEPDC1B promotes the localization of PI3K-C2β to cell-matrix adhesions, we induced adhesion recruitment of endogenous eGFP-PI3K-C2β in cells depleted of DEPDC1B. Pharmacological inhibition of PKN triggered the rapid accumulation of eGFP-PI3K-C2β at FAs in control cells, while a much weaker response was observed in DEPDC1B-depleted cells (Figures 4G and 4H). We conclude that DEPDC1B promotes the efficient recruitment of PI3K-C2β to cell-matrix adhesions to facilitate FA disassembly.

### PI3K-C2β-mediated synthesis of PI(3,4)P_2_ promotes focal adhesion disassembly

**Figure 5:**
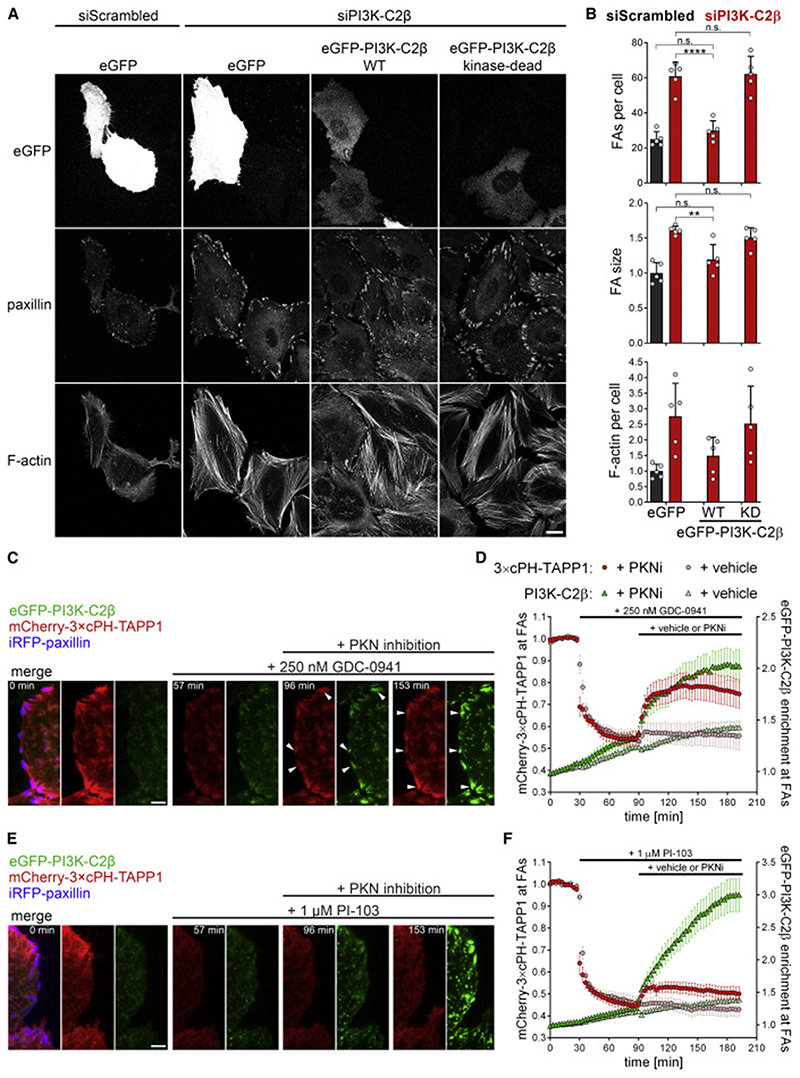
PI3K-C2β-mediated synthesis of PI(3,4)P_2_ triggers focal adhesion disassembly (A and B) Focal adhesion morphology in PI3K-C2β-depleted cells is reconstituted by re-expression of WT but not kinase-dead PI3K-C2β. SiRNA-treated cells were transfected with eGFP or either siRNA-resistant WT or kinase-dead eGFP-PI3K-C2β and stained for paxillin and F-actin (laser scanning confocal microscopy). (A) Scale bars, 20 μm. (B) Mean + SD from n = 5 independent experiments, two-way ANOVA with Tukey’s test. (C–F) The PI(3,4)P_2_ sensor 3 ×cPH-TAPP1 displays recruitment to cell-matrix adhesions in a PI3K-C2β-dependent manner. HeLa cells with endogenous eGFP-PI3K-C2β transiently expressing mCherry-3 × cPH-TAPP1 and iRFP-paxillin were imaged by TIRF microscopy at 3 min/frame. After 30 min, cells were treated with (C and D) 250 nM GDC-0941 (a pan-class I PI3K inhibitor sparing PI3K-C2β) or (E and F) 1-μM PI-103 (a pan-class I PI3K inhibitor hitting PI3K-C2β at IC_50_= 26 nM). After 90 min, cells were additionally subjected to PKN inhibition (1-μM PKC412 + 2-μM Cdk1/2 inhibitor III) to trigger recruitment of PI3K-C2β to adhesions. (C and E) Scale bars, 10 μm. (D and F) Enrichment of mCherry-3 × cPH-TAPP1 and eGFP-PI3K-C2β at focal adhesions was quantified as mean intensity at adhesions (as labeled by iRFP-paxillin in first frame) over mean intensity in the rest of the cell. Mean ± 95% confidence interval from n = 54 cells (GDC-0941 + vehicle), n = 62 cells (GDC-0941 + PKNi), n = 63 cells (PI-103 + PKNi) from three independent experiments and n = 44 cells (PI-103 + vehicle) from two independent experiments. KD, kinase-dead; FAs, focal adhesions.

The recruitment of PI3K-C2β to cell adhesions suggests a crucial function for local signaling lipids, i.e., 3-phosphoinositides in adhesion disassembly. To address this, we first probed whether the catalytic activity of PI3K-C2β is required in the regulation of FA disassembly. PI3K-C2β-selective kinase inhibitors are not available to date. We therefore expressed WT or KD eGFP-PI3K-C2β (Marat et al., 2017) in cells depleted of endogenous PI3K-C2β and monitored the effects on FAs. Whereas PI3K-C2β-WT restored the number of FAs as well as the size of individual adhesions and formation of stress fibers to those seen in control cells, mutant kinase-inactive PI3K-C2β was unable to rescue these phenotypes (Figures 5A and 5B). Of note, these findings are supported by our observations in MEFs from mice with KD PI3K-C2β (Alliouachene et al., 2015;Figures S1A–S1D, S2D, and S2E). These findings suggest a crucial contribution of PI3K-C2β-mediated synthesis of 3-phosphoinositide lipids in FA disassembly.

Class II PI3Ks display dual lipid substrate specificity, enabling them to produce either PtdIns(3)P or PtdIns(3,4)P_2_, likely depending on the substrate available in distinct subcellular compartments (Bilanges et al., 2019; Gulluni et al., 2019) and/or the presence of additional regulatory factors. The plasma membrane is known to be enriched in PtdIns(4)P (Hammond et al., 2012; Nakatsu et al., 2012) and to be 10-fold de-enriched in PtdIns compared with the total cellular PtdIns pool (Pemberton et al., 2020; Saheki et al., 2016; Zewe et al., 2020). Given that PI3K-C2β displays preference for PtdIns(4)P *in vitro* (Marat et al., 2017), we surmised PtdIns(3,4)P_2_ to be the likely product of PI3K-C2β at the plasma membrane. We tested this hypothesis by investigating whether recruitment of PI3K-C2β to FAs would cause detectable changes in PtdIns(3,4)P_2_ levels at these sites. To this end, we took advantage of a recently developed 3×cPH-TAPP1 biosensor for PtdIns(3,4)P_2_ (Goulden et al., 2019). Under basal culture conditions (i.e., in the presence of serum growth factors), a large fraction of plasma membrane PtdIns(3,4)P_2_ is generated by 5-phosphatase activity on PtdIns(3,4,5)P_3_ generated by class I PI3Ks (Goulden et al., 2019), thereby masking a potential contribution of PI3K-C2β to PtdIns(3,4)P_2_ synthesis. To uncover this class II PI3Kβ-dependent pool of PtdIns(3,4)P_2_, we treated cells with the pan-class I PI3K inhibitor GDC-0941, which has a reduced activity toward PI3K-C2β compared with the class I PI3Ks (IC_50_ concentrations for PI3Kα: 3 nM; PI3Kβ: 11 nM and PI3K-C2β: 670 nM) (Folkes et al., 2008). Cells carrying endogenous eGFP-PI3K-C2β transiently expressing mCherry-3×cPH-TAPP1 and iRFP-paxillin were imaged by TIRF micro-scopy. Treatment with 250 nM GDC-0941 caused an immediate drop in PtdIns(3,4)P_2_ levels (Figures 5C and 5D). Subsequent pharmacological inhibition of PKN led to a rapid increase of eGFP-PI3K-C2β at FAs, paralleled by a substantial rise in PtdIns(3,4)P_2_ at these sites (Figures 5C and 5D; Video S4). To challenge these experiments, we used the PI-103 pan-class I PI3K inhibitor, which also potently inhibits PI3K-C2β (IC_50_ = 26 nM) (Knight et al., 2006). Although recruitment of eGFP-PI3K-C2β to FAs was even more pronounced in cells treated with PI-103, this only elicited a weak PtdIns(3,4)P_2_ signal (Figures 5E and 5F).

As an independent approach to investigate the lipid product of PI3K-C2β at FAs, we capitalized on the observation that the substrate specificity of PI3Ks is encoded in their activation loop (Bilanges et al., 2019; Pirola et al., 2001; Posor et al., 2013). Mutation of the ^1228^KRDR^1231^ amino acid stretch to ^1228^KPLP^1231^ in PI3K-C2β (henceforth referred to as KPLP-mutant) has been shown to abrogate the formation of PtdIns(3,4)P_2_ without affecting the enzyme’s ability to synthesize PtdIns(3)P (Gozzelino, L. et al., Brain, 2022, personal communication). When expressed in cells depleted of endogenous PI3K-C2β, KPLP-mutant PI3K-C2β was unable to rescue the accumulation of FAs in these cells (Figures S5A and S5B), suggesting that the ability to form PtdIns(3,4)P_2_ is required for the role of PI3K-C2β in adhesion turnover. These combined data establish that PI3K-C2β locally synthesizes PtdIns(3,4)P_2_ to facilitate the disassembly of cell-matrix adhesions.

### Local PtdIns(3,4)P_2_ synthesis by PI3K-C2β downregulates RhoA via recruitment of the GAP ARAP3

**Figure 6:**
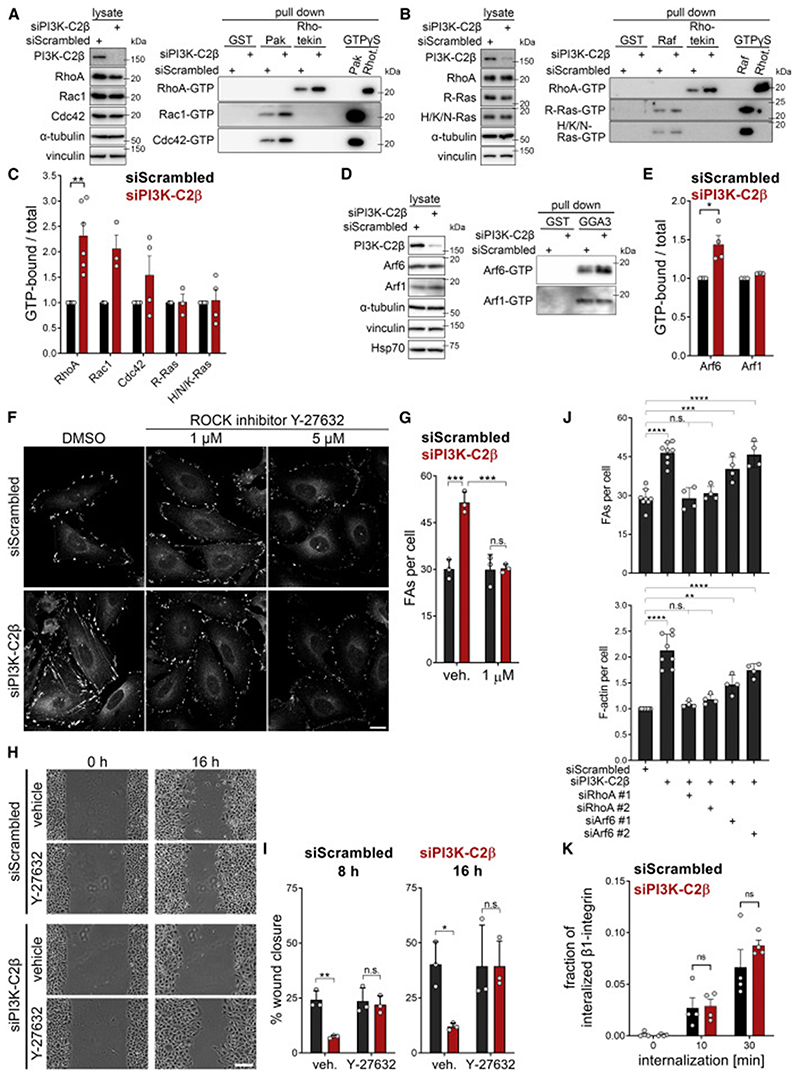
Increased RhoA-GTP levels underlie impaired adhesion turnover (A–E) Increased basal GTP levels of Rho- and Arf-family GTPases in cells depleted of PI3K-C2β as assayed by pull-downs using GST (control), GST-PAK-PBD, GST-Rhotekin-RBD, GST-Raf-RBD, or GST-GGA3^VHS-GAT^. GTP_γ_S-loaded lysates served as a positive control. Rhot., Rhotekin. (C and E) Densitometric quantification of immunoblots as shown in (A), (B), and (D). Mean + SEM from n = 6 (RhoA), n = 3 (Rac1, R-Ras, and Arf1) and n = 4 (Cdc42, H/N/K-Ras, and Arf6) independent experiments, one sample, two-tailed t test with hypothetical mean of 1.0. (F and G) Titration of the ROCK inhibitor Y-27632 on HeLa cells depleted of PI3K-C2β. Cells were treated with the indicated concentration of Y-27632 for 16-h overnight and stained for paxillin and imaged with a spinning disk confocal microscope. (F) Scale bars, 20 μm. (G) 1-μM Y-27632 rescues focal adhesion morphology defects in PI3K-C2β-depleted cells. Mean + SD from n = 3 independent experiments, two-way ANOVA with Tukey’s test. (H and I) Rescue of migration by low-dose Y-27632 ROCK inhibitor (1 μM) in PI3K-C2β-depleted cells in the scratch wound assay after 8 and 16h(in serum- and mitomycin-C-containing media). (H) Scale bars, 100 μm. (I) Mean + SD from n = 3 independent experiments, two-way ANOVA with Sidak’s test. (J) Co-depletion of RhoA, but not of Arf6, rescues focal adhesion morphology defects in PI3K-C2β-depleted cells. HeLa cells were siRNA treated as indicated (see also Figure S6C) and stained for paxillin (spinning disk confocal microscopy). Mean + SD from n = 4–8 independent experiments, one-way ANOVA with Dunnett’s test. (K) The endocytic rate of total β1-integrins is not affected by depletion of PI3K-C2β. The amount of β1-integrin internalized after different time points was measured using a surface biotinylation and streptavidin pull-down assay (see STAR Methods). Mean + SEM from n = 4 independent experiments, multiple unpaired t test with Welch’s correction. See also Figure S6. FAs, focal adhesions.

We next asked how PtdIns(3,4)P_2_ formation might facilitate the disassembly of cell-matrix adhesions. Small GTPases of the Rho- and Ras-families are critical regulators of the formation, maturation and stability of cell-matrix adhesions and associated cytoskeletal changes, and their activity state is known to be regulated by phosphoinositides (Lawson and Ridley, 2018; Ridley, 2015). We therefore assessed the steady-state activation levels of a panel of small GTPases upon PI3K-C2β depletion. RhoA-GTP levels were found to be increased in PI3K-C2β-depleted cells, with the levels of active Rac1-GTP, Cdc42-GTP, and Arf6-GTP being likewise increased but somewhat less affected (Figures 6A–6E). No changes were observed in the GTP levels of other small GTPases, including R-Ras, which has previously been linked with adhesion turnover (Kwong et al., 2003; Zhang et al., 1996; Li et al., 2022, or of H/N/K-Ras or Arf1 (Figures 6B–6E).

The accumulation of stress fibers and increased pMLC2^S19^ levels upon PI3K-C2β depletion (Figures 1D and 1E) are consistent with RhoA hyperactivation. We therefore analyzed whether altered RhoA activation is a cause or consequence of impaired cell-matrix adhesion turnover upon loss of PI3K-C2β-mediated PtdIns(3,4)P_2_-synthesis. To this end, we tested whether inhibition of ROCK, a key RhoA effector that promotes actomyosin contractility, rescues defective FA disassembly. Given that balanced RhoA activation is critical for cell-matrix adhesion dynamics, we probed the effects of partial ROCK inhibition by low doses of Y-27632, well below those known to achieve full kinase inhibition (Uehata et al., 1997; Watanabe et al., 2007). Treatment of cells with as little as 1 μM Y-27632 restored phosphorylation of the ROCK substrate MLC2 in PI3K-C2β-depleted cells to control levels (Figures S6A and S6B). Importantly, 1 μM Y-27632 fully rescued the number and size of FAs to control levels in PI3K-C2β-depleted cells, without significant effects in control-siRNA-treated cells (Figures 6F and 6G), and completely rescued cell migration in scratch wound assays (Figures 6H and 6I). To address the role of RhoA by an independent approach, we co-depleted cells of PI3K-C2β and either RhoA or Arf6. Only depletion of RhoA fully rescued the accumulation of FAs and of F-actin in PI3K-C2β-depleted cells (Figures 6J and S6C). Taken together, these findings show that impaired adhesion turnover in PI3K-C2β-depleted cells results from RhoA hyperactivation.

We next sought to understand the contribution of other pathways implicated in the regulation of FA disassembly to impaired adhesion turnover in PI3K-C2β-depleted cells. An important aspect of cell-matrix adhesion disassembly is the internalization of integrins via clathrin-mediated endocytosis (Ezratty et al., 2009). Indeed, PI3K-C2β has previously been reported to interact with clathrin (Wheeler and Domin, 2006) and the endocytic scaffolding protein intersectin (Das et al., 2007), yet no functional requirement in endocytosis has been described for PI3K-C2β thus far. Depletion of PI3K-C2β did not affect the endocytosis of β1-integrins as assessed by a surface biotinylation assay (Figures 6K and S7A), suggesting that defects in integrin internalization are not a direct cause underlying our observations.

PI3K-C2β has also been implicated in repressing mTORC1 activity upon cessation of growth factor signaling through the production of a local endolysosomal pool of PtdIns(3,4)P_2_ (Marat et al., 2017; Wallroth et al., 2019). In line with this, depletion of PI3K-C2β leads to hyperactivation of mTORC1 signaling, correlating with dispersion of a late endosomal/lysosomal compartment to the cell periphery (Marat et al., 2017; Wallroth et al., 2019). Recent reports of an mTORC1-Rho-ROCK signaling pathway (Peterson et al., 2015) and of a role for peripheral late endosomal translocation in FA turnover (Schiefermeier et al., 2014) raised the question of whether our observations may pertain to regulation of mTORC1 by PI3K-C2β. If impaired cell-matrix adhesion turnover in PI3K-C2β-depleted cells was linked to increased levels of mTORC1 activity, inhibition of mTORC1 by rapamycin should rescue accumulation of cell-matrix adhesions. However, overnight treatment with rapamycin did not alter the number or size of FAs in PI3K-C2β-depleted cells (Figures S7B and S7C). These data argue against altered mTORC1 activity to underlie impaired adhesion turnover in PI3K-C2β-depleted cells.

**Figure 7:**
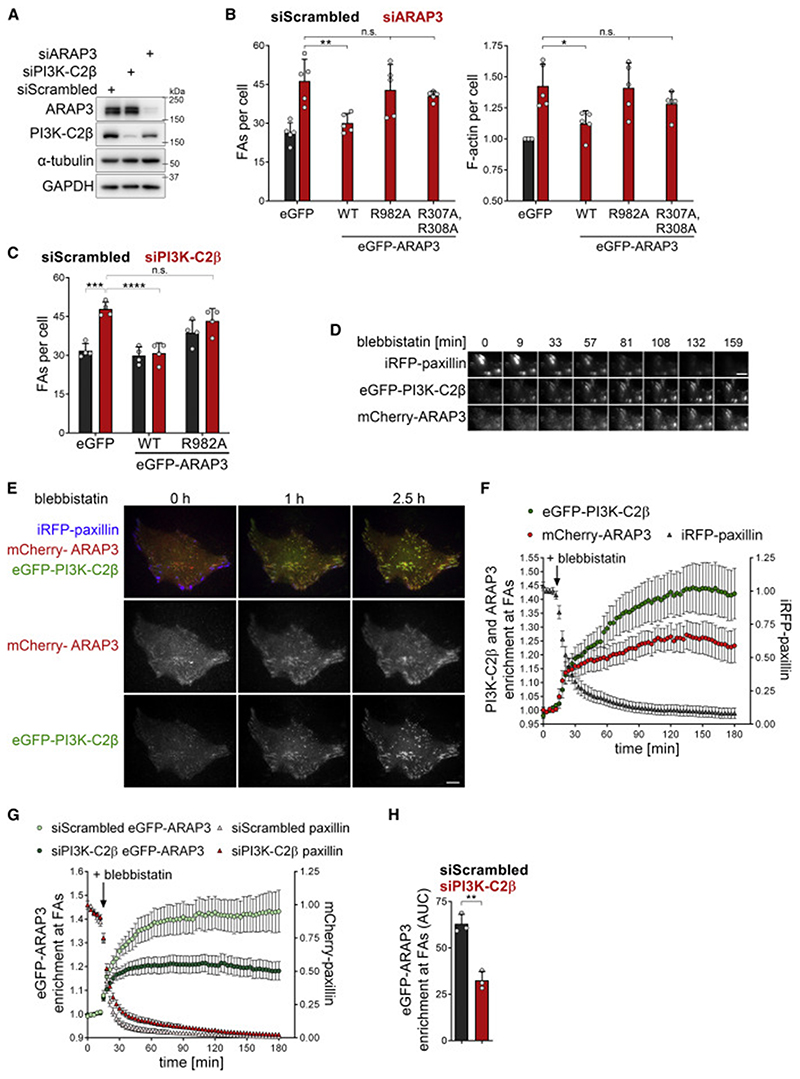
PI3K-C2β promotes the recruitment of the RhoA-GAP ARAP3 to disassembling adhesions (A) Immunoblot analysis of HeLa cells treated with siRNAs against PI3K-C2β or ARAP3. α-tubulin and GAPDH were detected as loading controls. Data shown are representative of five independent experiments. (B) Depletion of ARAP3 phenocopies accumulation of focal adhesions in PI3K-C2β-depleted cells. SiRNA-treated HeLa cells were transfected with eGFP or siRNA-resistant eGFP-ARAP WT, R982A (GAP-deficient mutant) or R307A,R308A (lipid-binding-deficient mutant). Cells were stained for paxillin and for F-actin (spinning disk confocal microscopy). Mean + SD from n = 5 independent experiments, one-way ANOVA with Holm-Sidak’s test. (C) Overexpression of WT but not GAP-deficient ARAP3 rescues accumulation of focal adhesions in PI3K-C2β-depleted cells. PI3K-C2β-depleted HeLa cells were transfected and analyzed as in (B). Mean + SD from n = 4 independent experiments, two-way ANOVA with Tukey’s test. (D–F) eGFP-PI3K-C2β and mCherry-ARAP3 display overlapping timing of recruitment to cell-matrix adhesions upon triggering adhesion disassembly by blebbistatin. HeLa cells with endogenous eGFP-PI3K-C2β transiently expressing mCherry-ARAP3 and iRFP-paxillin were imaged by TIRF microscopy at 3 min/ frame. After 5 frames, cells were treated with 25-μM blebbistatin. (D) Magnified view of four neighboring adhesions. Upon addition of blebbistatin and onset of adhesion disassembly, both eGFP-PI3K-C2β and mCherry-ARAP3 accumulate at the adhesion. Scale bars, 5 μm. (E) Scale bars, 10 μm. (F) Enrichment of mCherry-ARAP3 and eGFP-PI3K-C2β at focal adhesions was quantified as mean intensity at adhesions (as labeled by iRFP-paxillin at baseline) over mean intensity in rest of the cell. Mean ± 95% confidence interval from n = 37 cells from three independent experiments. (G and H) Blebbistatin-induced recruitment of ARAP3 is diminished upon depletion of PI3K-C2β. PI3K-C2β-depleted HeLa cells transiently expressing eGFP-ARAP3 and mCherry-paxillin were imaged and enrichment analyzed as in (D)–(F). (G) Mean ± 95% confidence interval from n = 81 cells (siScrambled) and n = 88 cells (siPI3K-C2β). (H) AUC from data shown in (G). Mean + SD from n = 3 independent experiments, unpaired two-tailed t test with Welch’s correction. See also Figure S7. FAs, focal adhesions.

We therefore followed the alternative hypothesis that local PtdIns(3,4)P_2_ synthesis by PI3K-C2β promotes adhesion disassembly via downregulation of RhoA-GTP. Rho family GTPase-activating proteins (GAPs) frequently harbor lipid-binding domains (Amin et al., 2016), suggesting that PI3K-C2β activity may directly regulate a RhoA-inactivating GAP at disassembling FAs. Among all Rho family GAPs hitherto described, only the PH domain-containing protein ARAP3 (Krugmann et al., 2002, 2004; Stacey et al., 2004) has been found to specifically bind to PtdIns(3,4)P_2_ (and, possibly, PtdIns(3,4,5)P_3_) (Craig et al., 2010; Krugmann et al., 2002) *in vitro* and to endosomes enriched in PtdIns(3,4)P_2_ in living cells (Lee et al., 2019). Moreover, the reported substrate specificity of ARAP3 matches precisely with the observed changes in GTP levels of RhoA, Rac1, Cdc42, and Arf6 in PI3K-C2β-depleted cells (Krugmann et al., 2002; Song et al.,1998). We therefore hypothesized that ARAP3 acts as a PtdIns(3,4)P_2_ effector of PI3K-C2β to direct cell-matrix adhesion disassembly. Indeed, ARAP3 depletion led to an accumulation of FAs and of F-actin stress fibers (Figures 7A and 7B), akin to PI3K-C2β loss.

If ARAP3 was an effector of PI3K-C2β activity, it would be predicted that regulation of adhesion turnover requires the ability of ARAP3 to bind phosphoinositides (i.e., PtdIns(3,4)P_2_) and to stimulate the activity of GTPases. We tested these predictions by conducting rescue experiments using WT, GAP-deficient (R982A) (Krugmann et al., 2004) or lipid-binding-deficient (R307A,R308A) eGFP-ARAP3 mutants (Krugmann et al., 2002). Whereas WT ARAP3 restored FA numbers and stress fiber formation to control levels, re-expression of GAP-deficient or lipid-binding-deficient ARAP3 mutants failed to rescue these phenotypes (Figures 7A and 7B). Moreover, overexpression of WT but not GAP-deficient ARAP3 in PI3K-C2β-depleted cells was sufficient to reduce the number of FAs (Figure 7C). These data suggest that PI3K-C2β may recruit ARAP3 to disassembling adhesion sites. To probe this hypothesis further, we monitored the local nanoscale recruitment of both proteins in live cells. To this aim, cells were treated with blebbistatin to induce FA disassembly. eGFP-PI3K-C2β and mCherry-ARAP3 were enriched at disassembling adhesions and displayed overlapping time courses of recruitment (Figures 7D–7F). Importantly, recruitment of ARAP3 to FAs was attenuated in PI3K-C2β-depleted cells (Figures 7G and 7H).

These collective data establish ARAP3 as a PtdIns(3,4)P_2_ effector of PI3K-C2β in cell-matrix adhesion disassembly.

## Discussion

Despite a detailed understanding of the proteomic changes associated with cell-matrix adhesion turnover (Revach et al., 2020), it has remained unclear how the composition of the membrane affects these dynamics. Here, we unravel a pathway that links signaling input into the PKN2 Ser/Thr kinase (Wallroth et al., 2019) to the local formation of PtdIns(3,4)P_2_ to trigger disassembly of FAs. Our findings suggest a model whereby the lack of stimulatory input into PKN2, e.g., as a consequence of reduced growth factor signaling, triggers the accumulation of PI3K-C2β at FAs through association with DEPDC1B, a poorly characterized protein implicated in adhesion disassembly (Marchesi et al., 2014). This results in the formation of PtdIns(3,4)P_2_ by PI3K-C2β at FAs, promoting recruitment of the Rho-GAP ARAP3 and downregulation of RhoA activity, tipping the balance toward adhesion disassembly. Our results further imply that whereas formation and stabilization of FAs are driven by PtdIns(4,5)P_2_ synthesis (Di Paolo et al., 2002; Legate et al., 2011), a switch to PtdIns(3,4)P_2_ generation then facilitates adhesion disassembly via inactivation of RhoA. The local conversion of phosphoinositide lipids (also called “lipid switch”) isan emerging theme in endo-exocytic membrane traffic (Wang et al., 2019) as well as the regulation of lysosomal membrane dynamics (Ebner et al., 2019). In this context, PI3K-C2β appears to function as a mediator of the cellular response to starvation by producing local pools of PtdIns(3,4)P_2_ to confer repression of mTORC1 on lysosomes (as reported by us earlier (Marat et al., 2017; Wallroth et al., 2019)) and to concomitantly initiate turnover of cell-matrix adhesions at the plasma membrane (this study). Interestingly, FAs may serve as mTORC1 activation hubs (Rabanal-Ruiz et al., 2021), further integrating the function of PI3K-C2β in FA disassembly identified here with its role in the cellular response to starvation.

The PtdIns(4)P used by PI3K-C2β at FAs could derive from the plasma membrane PtdIns(4)P-pool, which is known to largely depend on PI4KIIIα (Nakatsu et al., 2012) and/or could require local formation from PtdIns(4,5)P_2_ by a 5′-phosphatase such as SHIP2, which indeed has been implicated in FA disassembly (Fukumoto et al., 2017). Counter-transport of PtdIns(4)P and phosphatidylcholine from the endoplasmic reticulum by the lipid transfer protein ORP3 has recently been reported to facilitate FA disassembly (D’Souza et al., 2020), suggesting that PtdIns(4)P may thus contribute to adhesion disassembly by serving as a substrate for both PI3K-C2β and ORP3.

Release from inhibitory 14-3-3 proteins enables PI3K-C2β to associate with different cellular organelles, mediated by Rab7/ Raptor on endolysosomes (Marat et al., 2017; Wallroth et al., 2019) and DEPDC1B at FAs (Figure 4). Unlike reported for DEPDC1B (Marchesi et al., 2014), we found no significantly delayed mitotic entry in PI3K-C2β-depleted HeLa cells (Figures 2H and S2F), and indeed observed pronounced accumulation of PI3K-C2β at FAs in cells preparing for cytokinesis well before detachment (Figures 2F and 2G). This may reflect known differences in the disassembly of cell-matrix adhesions in interphase and mitosis (Dix et al., 2018; Ramkumar and Baum, 2016), with a more prominent role of PI3K-C2β in the turnover of adhesions during interphase.

Not only cell-matrix but also cell-cell adhesions are closely connected with the actomyosin cytoskeleton, with balanced RhoA activation being similarly important for maintenance and stabilization of adherens junctions as it is for cell-matrix adhesions (Citi et al., 2014; Sahai and Marshall, 2002; Terry et al., 2011). Interestingly, a recent study reports that inhibition of PI3K-C2β strengthens the vascular barrier by stabilizing VE-cad-herin at adherens junctions (Anquetil et al., 2021). ARAP3 is also expressed in endothelia (Van Buul et al., 2014) and essential for developmental angiogenesis (Gambardella et al., 2010), raising the possibility of a more widespread relevance of our findings in the regulation of cellular adhesion. Future research will have to clarify the roles of PI3K-C2β in regulating both cell-matrix and cell-cell adhesion in epithelia, which are of high relevance not only for cardiovascular disorders (Anquetil et al., 2021) but also cancer metastasis and organ development (Pandya et al., 2017).

### Limitations of the study

An interesting observation is that enrichment of PI3K-C2β persists at sites of disassembled adhesions, well after adhesion markers such as paxillin have dissociated from the adhesive structures (Figures 2E–2G). This could reflect a requirement for sustained local RhoA inactivation to ensure continued adhesion dismantling despite the presence of activated integrins, preventing re-growth of the adhesion. A non-exclusive alternative explanation, which we have not explored in depth in this study, is that PI3K-C2β might facilitate the internalization of integrins (Ezratty et al., 2009), an endocytic cargo that is known to cause increased resistance to internalization (Baschieri et al., 2018). As we did not observe changes in the internalization of total β1-integrins (Figures 6K and S7A), a severe effect of PI3K-C2β depletion on the endocytic process itself appears unlikely. However, we cannot exclude a specific role of PI3K-C2β in the endocytosis of active β1-integrins.

The implications of our findings for tissue development and maintenance (Pandya et al., 2017; van der Stoel et al., 2020) remain to be explored. FAs in migrating epithelial sheets are distributed asymmetrically, with leader cells at the migratory front forming stronger matrix adhesions and generating contractile forces to pull the follower cells (De Pascalis and Etienne-Manneville, 2017). Our observations suggest that during migration along a growth factor gradient, lower PKN2 activity in follower cells might promote PI3K-C2β-dependent FA turnover. This would weaken cell-matrix adhesion to permit passive cell movement, whereas high growth factor input at the migratory front would keep PI3K-C2β inactive, thereby ensuring stable matrix adhesion to generate the necessary contractile force.

### Star⋆Methods

Detailed methods are provided in the online version of this paper and include the following:

- KEY RESOURCES TABLE
- RESOURCE AVAILABILITY
  ○ Lead contact
  ○ Materials availability
  ○ Data and code availability
- EXPERIMENTAL MODEL AND SUBJECT DETAILS
- METHOD DETAILS
  ○ Plasmids
  ○ Antibodies
  ○ siRNAs
  ○ Inhibitors and chemicals
  ○ DNA and siRNA transfection
  ○ Immunocytochemistry and confocal microscopy
  ○ TIRF imaging
  ○ Cell extracts and immunoblotting
  ○ Immunoprecipitations
  ○ GTPase pull-down assays
  ○ Scratch wound migration assay
  ○ Nocodazole washout assay
  ○ Yeast-2-hybrid screen
  ○ RNA isolation and RT-qPCR
  ○ Genome-editing using CRISPR/Cas9
  ○ Generation of mouse embryonic fibroblasts
  ○ Surface biotinylation b1-integrin endocytosis
  ○ Measurement of mitotic rounding
- QUANTIFICATION AND STATISTICAL ANALYSIS
  ○ Image analysis
  ○ Statistics

## Star⋆Methods

### Key Resources Table

**Table:** 

REAGENT or RESOURCE	SOURCE	IDENTIFIER
Antibodies				
Mouse monoclonal anti-paxillin	BD Bioscience	Cat# 610051; RRID: AB_397463
Mouse monoclonal anti-phospho-MLC2 S19	Cell Signaling Technology	Cat# 3675; RRID: AB_2250969
Rabbit polyclonal anti-total MLC2	Cell Signaling Technology	Cat# 3672; RRID: AB_10692513
Mouse monoclonal anti-α-tubulin	Merck (Sigma-Aldrich)	Cat# T5168; RRID: AB_477579
Mouse monoclonal anti-c-myc	Abcam	Cat# ab32; RRID: AB_303599
Mouse monoclonal anti-FLAG	Merck (Sigma-Aldrich)	Cat# F-3165; RRID: AB_259529
Rabbit polyclonal anti-mouse IgG	Merck (Sigma-Aldrich)	Cat# M-7023; RRID: AB_260634
Rabbit polyclonal anti-DEPDC1B	Biorbyt	Cat# orb183064
Rabbit polyclonal anti-DEPDC1B	MyBiosource	Cat# mbs154376
Mouse monoclonal anti-DEPDC1B	Merck (Sigma-Aldrich)	Cat# SAB1403301; RRID: AB_10738419
Mouse monoclonal anti-β-actin	Merck (Sigma-Aldrich)	Cat# A1978; RRID: AB_476692
Mouse monoclonal anti-PI3K-C2β	BD Bioscience	Cat# 611342; RRID: AB_398864
Mouse monoclonal anti-Talin	Merck (Sigma-Aldrich)	Cat# T3287; RRID: AB_477572
Rabbit polyclonal anti-eGFP	Abcam	Cat# ab6556; RRID: AB_305564
Rabbit polyclonal anti-Erk1/2	Cell Signaling Technology	Cat# 9102; RRID: AB_330744
Mouse monoclonal anti-14-3-3 (pan)	Santa Cruz Biotechnology	Cat# sc-1657; RRID: AB_626618
Rabbit monoclonal anti-RhoA	Cell Signaling Technology	Cat# 2117; RRID: AB_10693922
Mouse monoclonal anti-Rac1	Merck-Millipore	Cat# 05-389; RRID: AB_309712
Rabbit polyclonal anti-Cdc42	Cell Signaling Technology	Cat# 2462; RRID: AB_2078085
Mouse monoclonal anti-vinculin	Merck (Sigma-Aldrich)	Cat# V9131; RRID: AB_477629
Rabbit polyclonal anti-R-Ras	Cell Signaling Technology	Cat# 8446; RRID: AB_10838402
Mouse monoclonal anti-H/K/N-Ras	BD Bioscience	Cat# 610001; RRID: AB_397424
Rabbit polyclonal anti-ARAP3	Novus	Cat# NBP1-84541; RRID: AB_11007042
Mouse monoclonal anti-GAPDH	Abcam	Cat# ab8245; RRID: AB_2107448
Rabbit polyclonal anti-phospho-FAK Y397	Cell Signaling Technology	Cat# 3283; RRID: AB_2173659
Mouse monoclonal anti-total FAK	BD Bioscience	Cat# 610087; RRID: AB_397494
Rabbit polyclonal anti-phospho-paxillin Y31	Abcam	Cat# ab32115; RRID: AB_777116
Rabbit polyclonal anti-Arf6 (C-term.)	(Song et al., 1998)	N/A
Mouse monoclonal anti-Arf1	Abcam	Cat# ab18108; RRID: AB_444251
Mouse monoclonal anti-Hsp70	Invitrogen (ThermoFischer)	Cat# MA3-006; RRID: AB_325454
Mouse monoclonal anti-β1-integrin	BD Bioscience	Cat# 610467; RRID: AB_2128060
Rabbit polyclonal anti-Transferrin receptor	Merck (Sigma-Aldrich)	Cat# HPA028598; RRID: AB_10601599
Chemicals, peptides, and recombinant proteins		
Phalloidin-AlexaFluor647	ThermoFisher	Cat# A22287
Nocodazole	Merck (Sigma-Aldrich)	Cat# M1404
para-Nitroblebbistatin	Axol	Cat# ax494693
PKC412	Selleck Chemicals	Cat# S8064
Cdk1/2 inhibitor III	EMD Millipore	Cat# S217714
GDC-0941	ApexBio via Stratech	Cat# A3432-APE
PI-103	EMD Millipore	Cat# 528100
Y-27632	Cayman Chemical	Cat# 10005583
Rapamycin	Calbiochem	Cat# 553210
Mitomycin C	Merck (Sigma-Aldrich)	Cat# M4287
GTP_γ_S	Merck (Sigma-Aldrich)	Cat# G8634
FuGENE HD transfection reagent	Promega	Cat# E2311
FuGENE 6 transfection reagent	Promega	Cat# E2691
DharmaFECT 1 Transfection Reagent	Horizon Discovery	Cat# T-2001-02
JetPRIME Transfection Reagent	Polyplus	Cat# 101000046
DharmaFECT Duo Transfection Reagent	Horizon Discovery	Cat# T-2010-02
Cas9 nuclease mRNA	Horizon Discovery	Cat#CAS11859
Edit-R tracrRNA	Horizon Discovery	Cat# U-002005-05
Matrigel	Corning	Cat# 354263
ProLong Gold Antifade Mountant	ThermoFisher	Cat# P10144
GFP-Trap Magnetic Agarose	Chromotek	Cat# gtma-20
Binding Control Magnetic Agarose Beads	Chromotek	Cat# bmab-20
PAK-PBD beads	Cytoskeleton	Cat# PAK02-A
Sulfo-NHS-SS-Biotin	ThermoFisher	Cat# 21331
Critical commercial assays		
Direct-zol RNA Miniprep	Zymo Research	Cat# R2051
High Capacity cDNA RT kit	Applied Biosystems	Cat# 10400745
QIAprep Spin Miniprep Kit	Qiagen	Cat# 27106
QIAquick PCR & Gel Cleanup Kit	Qiagen	Cat# 28506
QuikChange II XL Site-Directed Mutagenesis Kit	Agilent	Cat# 200523
Experimental models: Cell lines		
HeLa	ATCC	Cat# CCL-2, RRID: CVCL_0030
HeLa Kyoto, eGFP-knock-in for endogenous eGFP-PI3K-C2β expression	(Wallroth et al., 2019)	N/A
HEK293T, eGFP-knock-in for endogenous eGFP-PI3K-C2β expression	(Marat et al., 2017)	N/A
Mouse embryonic fibroblasts with PI3K-C2β D1212A mutation	(Alliouachene et al., 2015)	N/A
MDA-MB-231	ATCC	Cat# HTB-26; RRID: CVCL_0062
Oligonucleotides		
DEPDC1B ON-Targetplus SmartPool	Horizon Discovery	Cat# L-013830-00-0005
RhoA ON-Targetplus SmartPool (siRNA RhoA #2)	Horizon Discovery	Cat# L-003860-00-0005
Arf6 ON-Targetplus SmartPool (siRNA Arf6 #1)	Horizon Discovery	Cat# L-004008-00-0005
All other siRNAs: see Table S2	Eurofins / Sigma	N/A
DEPDC1B qPCR primers: see Table S2	Eurofins	N/A
Recombinant DNA		
Paxillin-eGFP	Addgene	Cat# 15233
pmCherry-paxillin	Addgene	Cat# 50526
pNES-iRFP-C1	Addgene	Cat# 116863
pmiRFP-paxillin	This study	N/A
eGFP-PI3K-C2β WT	(Marat et al., 2017)	N/A
eGFP-PI3K-C2β kinase dead	(Marat et al., 2017)	N/A
eGFP-PI3K-C2βWT T279A	This study	N/A
eGFP-PI3K-C2β KPLP-mutant	This study	N/A
6 × myc-mousePI3K-C2 β	This study	N/A
eGFP-DEPDC1B	This study	N/A
mCherry-3 × cPH-TAPP1	Addgene	Cat# 116862
GST-hRaf-RBD (1-147)	P. Rodriguez-Viciana (UCL Cancer Institute, UK)	N/A
GST-hRhotekin-RBD (7-89)	P. Rodriguez-Viciana (UCL Cancer Institute, UK)	N/A
eGFP-ARAP3 WT	Addgene	Cat# 39484
eGFP-ARAP3 R982A	Addgene	Cat# 39487
eGFP-ARAP3 R307A,R308A	Addgene	Cat# 39486
mCherrry-ARAP3	This study	N/A
eGFP-ARAP3 WT siRNA-resistant	This study	N/A
eGFP-ARAP3 R982A siRNA-resistant	This study	N/A
eGFP-ARAP3 R307A,R308A siRNA-resistant	This study	N/A
Software and algorithms		
ImageJ	(Schindelin et al., 2012)	RRID:SCR_003070
GraphPad Prism 9	GraphPad Software	RRID:SCR_002798

## Resource Availability

### Lead contact

Further information and requests for resources and reagents should be directed to and will be fulfilled by the lead contact, York Posor (posor@fmp-berlin.de).

### Materials availability

Plasmids generated in this study will be available upon request with a complete Material Transfer Agreement or will be available through Addgene.

## Experimental Model and Subject Details

HeLa, MDA-MB-231 and HEK293T cells were obtained from the American Type Culture Collection (ATCC). Generation of genome-engineered HeLa Kyoto and HEK293T cells with an eGFP-coding sequence inserted following the start-codon of endogenous PI3K-C2β has been described before (Marat et al., 2017; Wallroth et al., 2019). Mouse embryonic fibroblasts from E13.5 embryos of wild-type or *Pikc3c2*β^D1212A/D1212A^ mice were obtained as described earlier (Alliouachene et al., 2015). Primary MEFs were immortalized by stable transduction with a p53-targeting shRNA. All cells were cultured in DMEM with 4.5 g/L glucose, 10% fetal bovine serum and 100 U/mL penicillin/streptomycin at 37°C and 5% CO_2_. All cell lines were routinely tested for mycoplasma to ensure contamination-free cultures.

## Method Details

### Plasmids

Chicken Paxillin-eGFP was a gift from Rick Horwitz (Addgene #15233). Human pmCherry-paxillin was a gift from Kenneth Yamada (Addgene #50526). pmiRFP-paxillin was generated by subcloning a *Kpn*I-*Nhe*I fragment from MB3_pNES-iRFP-C1, a gift from Gerry Hammond (University of Pittsburgh, USA), into pmCherry-paxillin to replace mCherry with iRFP. Human eGFP-PI3K-C2βWT and kinase-dead with siRNA-resistant mutations were described earlier (Marat et al., 2017). The T279A and ^1228^KRDR^1231^ to ^1228^KPLP^1231^ mutations in eGFP-PI3K-C2β were introduced by site-directed mutagenesis using the QuikChangeII kit (Agilent) according to the manufacturer’s instructions. 6×myc-mPI3K-C2β was generated by cloning the mouse PI3K-C2β coding sequence via *EcoR*I / *Not*I into a pcDNA3.1(+)-based vector with a 6×myc-tag in between the *Kpn*I and *BamH*I sites. eGFP-DEPDC1B was generated by cloning a HeLa cell cDNA-amplified human DEPDC1B coding sequence via *BamH*I / *Not*I into a pcDNA3.1(+)-based vector with an eGFP-tag in between the *Kpn*I and *BamH*I sites. The PI(3,4)P_2_ sensor mCherry-3×cPH-TAPP1 was a gift from Gerry Hammond. Bacterial expression vectors for GST-hRaf-RBD (1-147) and GST-hRhotekin-RBD (7-89) were kindly provided by Pablo Rodriguez-Viciana (UCL Cancer Institute, United Kingdom). Human eGFP-ARAP3 WT (Addgene #39484), R982A (Addgene #39487) and R307A,R308A (Addgene #39486) were gifts from Sonja Vermeren. mCherrry-ARAP3 was generated by inserting a PCR-amplified mCherry-tag in between the *Age*I and *EcoRI* sites in eGFP-ARAP3 WT. The siRNA-resistant constructs of eGFP-ARAP3 were obtained through gene synthesis (Genewiz) of a *Not*I (nucleotide position 2797 in the ARAP3 coding sequence) to *Mfe*I (position 6059) fragment containing a) four silent mutations in each of the four siRNA target sequences and b) destroyed *EcoR*I and *Sac*II sites in the 3’-MCS. This fragment was subsequently cloned into eGFP-ARAP3 WT. The R982A (via *Spe*I to *Sac*II) and R307A,R308A (via *EcoR*I to *Not*I) mutations in siRNA-resistant eGFP-ARAP3 were generated by subcloning fragments into the siRNA-resistant eGFP-ARAP3 WT construct.

### Antibodies

Primary antibodies used in this study are listed in Table S1. Secondary HRP-coupled antibodies against mouse or rabbit IgG for immunoblotting (GE Healthcare) were used at 1:2000 – 1:5000 dilution. Secondary antibodies for immunocytochemistry were coupled to AlexaFluor488, -568 or -647 dyes (ThermoFisher Scientific) and were used at 1:400 dilution.

### siRNAs

siRNA oligonucleotides are listed in Table S2. Unless indicated otherwise, siRNA oligonucleotides were synthesized by Eurofins Genomics with 3’-dTdT overhangs and no other modifications. For DEPDC1B and ARAP3 knockdowns, pools of four siRNAs were used.

### Inhibitors and chemicals

Small molecule inhibitors and chemicals used in this study are listed in Table S3.

### DNA and siRNA transfection

For plasmid DNA transfection, cells were transfected with FugeneHD or Fugene6 (Promega) according to the manufacturer’s instructions. Per 6-well of HeLa cells, a total of 3.2 μg of plasmid DNA was used with 10 μL of FugeneHD or 8 μL Fugene6. Per 10 cm dish of HEK293 cells, a total of 17 μg of plasmid DNA was used with 51 μL of FugeneHD. Cells were generally analysed 24 h after transfection.

For depletion of PI3K-C2β or ARAP3, HeLa cells were transfected with siRNAs using Dharmafect 1 (Horizon Discovery) or jetPrime (Polyplus Transfection) according to the manufacturer’s instructions. Per 6-well, 0.2 nmol of siRNAs and 4 μL of Dharmafect 1 or 4 – 6 μL of jetPrime were used (this was scaled according to growth area for other culture dish sizes). Cells were analysed 72 h after siRNA transfection. In MDA-MB-231 cells, knockdowns were performed using jetPrime. Per 6-well, 0.2 nmol of siRNAs and 6 μL of jetPrime were used according to the manufacturer’s instructions.

For depletion of DEPDC1B, HeLa cells were transfected with jetPrime. Cells were seeded and simultaneously transfected (reverse transfection) on day 1, transfected again on day 2 and analysed on day 4. Per 12-well, 0.055 nmol of siRNAs and 3 μL of jetPrime were used.

Co-depletion of PI3K-C2β and RhoA or Arf6 required an extended knock-down protocol. HeLa cells were seeded on day 0, transfected using jetPrime on day 1 and day 3 and analysed 96 h after the first siRNA transfection. Per 6-well, 0.1 nmol of each siRNA and 4 μL jetPrime were used.

### Immunocytochemistry and confocal microscopy

HeLa and MDA-MB-231 cells were seeded on glass coverslips coated with Matrigel (Corning; Matrigel HC diluted 1:50 in OptiMEM, 1 h at 37°C). Cells were fixed in 4% formaldehyde in PBS for 15 min at room temperature. To reduce cytoplasmic paxillin background, cells were instead fixed in 4% formaldehyde + 0.15% Triton X-100 in PBS for 5 min at room temperature, followed by fixation in 4% formaldehyde in PBS for another 10 min. Cells were washed three times with PBS and permeabilized in 0.2% Triton X-100 in PBS at room temperature and then blocked in 3% BSA in PBS for 1 h. Primary antibodies were diluted in 3% BSA in PBS and incubated with the cells for 2 h at room temperature or 4°C overnight. Coverslips were washed three times in PBS and incubated with secondary antibodies and phalloidin (if applicable) diluted in 3% BSA in PBS for 1 h at room temperature. Coverslips were washed and mounted on glass slides using ProLong Gold (ThermoFisher Scientific).

Laser scanning confocal microscopy was performed using Zeiss LSM700, LSM780 or LSM880 confocal microscopes. For spinning disk confocal microscopy, we used a combined spinning disk-TIRF setup (3i Intelligent Imaging Innovations, see under TIRF imaging for more details).

### TIRF imaging

For TIRF imaging of live cells, HeLa cells were seeded on Matrigel-coated chambered coverglass 8-well slides (ThermoFisher Scientific # 155409PK or ibidi # 80827) 48 h before imaging. Before imaging, medium was changed to 300 μL per well of Fluorobrite DMEM (ThermoFisher Scientific) with 5% fetal bovine serum and 100 U/mL penicillin / streptomycin. All experiments were performed at 37°C and 5% CO_2_.

TIRF microscopy was performed using a combined spinning disk-TIRF setup (Zeiss - 3i Marianas SDC CSU-W1 system, 3i Intelligent Imaging Innovations) equipped with a VectorTIRF unit, Zeiss DefiniteFocus2, Prime 95B sCMOS cameras (Teledyne Photometrics), Zeiss Plan-Apochromat 63x/1.46 oil DIC M27 and Zeiss Plan-Apochromat 100x/1.46 oil DIC M27 objectives. The TIRF angle was individually adjusted for every experiment to ensure sufficiently shallow illumination depth. Per experiment, up to 50 positions across two neighbouring wells of the 8-well slide were imaged in parallel, allowing direct comparison of two treatments. For triple colour TIRF imaging (eGFP, mCherry, iRFP) we used adjusted z-offsets for each channel and each position to compensate for chromatic aberration-induced focus differences.

### Cell extracts and immunoblotting

Cultured cells were washed once in cold PBS and scraped in lysis buffer (20 mM Hepes pH 7.4, 100 mM KCl, 2 mM MgCl_2_, 1% Triton X-100, 1% protease and phosphatase inhibitor cocktails [Calbiochem]). After 5-15 min on ice, lysates were centrifuged at 20,800 × g at 4°C for 3 min and cleared supernatants were collected. Protein concentration was determined by Bradford assay and samples were adjusted to 1× Laemmli sample buffer, heated to 95°C for 5 min and stored at -20°C. Samples were resolved by SDS-PAGE and transferred to nitrocellulose membranes using wet blotting (Bio-Rad) or dry blotting (iBlot2, ThermoFisher Scientific). Primary antibodies (see Table S1) were diluted in 3% BSA, 0.02% NaN_3_ in TBS and incubated on the blots shaking at 4°C overnight or for two days. Secondary HRP-conjugated antibodies were diluted in 3% milk powder in TBS and incubated on the blots for 1-2 h at room temperature. Chemiluminescence was detected with a LAS4000 digital imaging system (GE Healthcare).

### Immunoprecipitations

For immunoprecipitation from transiently eGFP-DEPDC1B and 6×myc-PI3K-C2β expressing HEK293T cells, cells on 10 cm dishes were harvested 48 h after transfection. Cells were washed once in ice-cold PBS and collected in lysis buffer (20 mM HEPES pH 7.4, 100 mM KCl, 2 mM MgCl_2_, 1% Triton X-100, 1% protease and phosphatase inhibitor cocktails [Calbiochem]). Crude lysates were centrifuged at 20,800×g at 4°C for 10 min, cleared lysates were collected and protein concentration was determined by Bradford assay. Cleared lysates were incubated with 5 μg of primary antibodies, [i.) anti-c-Myc or anti-Flag as a control, ii.) anti-DEPDC1B (Biorbyt) or anti-mouse IgG as a control] bound to magnetic protein A or G beads rotating at 4°C overnight. Beads were washed five times in lysis buffer and bound proteins were eluted using 1 × Laemmli sample buffer.

For immunoprecipitation from genome-engineered HEK293T cells with endogenous eGFP-PI3K-C2β, cells were seeded onto 10 cm dishes coated with poly-L-lysine (Sigma-Aldrich). Cells were washed once in ice-cold PBS and collected in IP buffer (20 mM HEPES pH 7.4, 150 mM NaCl, 0.05% saponin, 1% protease and phosphatase inhibitor cocktails [Calbiochem]). Crude lysates were centrifuged at 20,800× g at 4°C for 10 min, cleared lysates were collected and protein concentration was determined by Bradford assay. Cleared lysates were incubated with control or GFP-trap magnetic beads (ChromoTek) for 4 h rotating at 4°C. Beads were washed five times in IP buffer and bound proteins were eluted using 1 × Laemmli sample buffer.

### GTPase pull-down assays

GST-hRaf-RBD (1-147), GST-hRhotekin-RBD (7-89) and GST-hGGA3-VHS-GAT (1-313) were expressed in *E. coli* and purified by batch method on GSH-agarose (ThermoFisher Scientific). Per pull-down, we used 80 μg of fusion protein. GST-Pak-PBD beads were obtained from Cytoskeleton; per pull-down, 50 μg of fusion protein were used. As a positive control, we used GTP_γ_S-loaded HeLa extract (see below) prepared by sequentially adjusting the cleared lysate to 15 mM EDTA, adding to 0.2 mM GTP_γ_S (Sigma-Aldrich) and incubating at 30°C for 15 min, transferring to ice and adjusting to 60 mM MgCl_2_.

SiRNA-treated HeLa cells (one 15 cm dish per pull-down sample) were washed once with ice-cold PBS and collected in GTPase lysis buffer (G-LB: 50 mM Tris pH 7.2, 500 mM NaCl, 10 mM MgCl_2_, 1% Triton X-100, 1% phosphatase inhibitor cocktail [Calbiochem] + one tablet complete mini protease inhibitor, EDTA-free [Merck] per 7 mL). Crude lysates were immediately centrifuged at 20,800×g at 4°C for 2 min and cleared lysates incubated with GST-hRaf-RBD, GST-hRhotekin-RBD or GST-Pak-PBD beads rotating at 4°C for 1 h. Unbound supernatant was discarded, beads were sucked dry using a needle and washed once in 25 mM Tris pH 7.2, 150 mM NaCl, 10 mM MgCl_2_, 1% Triton X-100. Bound proteins were eluted using 1 × Laemmli sample buffer.

### Scratch wound migration assay

siRNA-treated HeLa cells were seeded to Matrigel-coated 6-wells and grown to a confluent monolayer. A scratch wound was introduced using a P200 pipette tip followed by washing cells twice with PBS and addition of fresh culture medium with 10% FBS containing 1 μg/mL mitomycin C (to block cell proliferation and thereby to assess migration independently of proliferation). Wound closure was documented at 0 h, 8 h and 24 h using an EVOS DIC microscope (ThermoFisher Scientific) and percentage closure of the initial wound was quantified using ImageJ.

For scratch wound assays with immortalized MEFs, cells were seeded in non-coated 6-wells and grown to confluence. For PDGF-stimulated wound closure, cells were starved of serum overnight. A scratch wound was made as described above and cells were allowed to migrate in DMEM + 50 ng/mL PDGF (no serum) or in full medium with 10% FBS. Proliferation was inhibited using 10 μg/mL mitomycin C.

### Nocodazole washout assay

siRNA-treated HeLa cells seeded on Matrigel-coated glass coverslips were treated with 10 μM nocodazole for 2 h to depolymerize microtubules and inhibit adhesion disassembly. Synchronised adhesion disassembly was triggered by removal of nocodazole with two washes in PBS, and cells were incubated in full medium for 15 min to allow adhesion disassembly to proceed. Cells were fixed using 4% formaldehyde for 15-20 min and stained for paxillin and α-tubulin.

For nocodazole washout in MEFs, cells on uncoated glass coverslips were serum-starved overnight and treated with 10 μM nocodazole for 2 h. After 10 minutes of nocodazole washout, cells were fixed and stained for paxillin or α-tubulin.

### Yeast-2-hybrid screen

A genome-wide ULTImate yeast-2-hybrid screen was performed by Hybrigenics Services (Paris, France) with a bait construct encoding amino acids 1-615 of human PI3K-C2β cloned in pB29 (N-bait-LexA-C fusion) against a human placenta_RP5 complementary cDNA Gal4-activating domain-fusion library.

### RNA isolation and RT-qPCR

HeLa cells were grown in 6-wells and RNA extracted using the Direct-zol RNA MiniPrep kit (Zymo) according to the manufacturer’s instructions. cDNA was generated by reverse transcription of 200 - 250 ng of purified RNA using the High Capacity cDNA RT kit (Applied Biosystems). Quantitative PCR reactions were run using the PowerUP SYBR Green Master Mix (Applied Biosystems) on a QuantStudio6 Real-Time PCR System (Applied Biosystems). Mean C_t_ values were calculated from technical replicates and gene expression was calculated using a standard curve from serially diluted pooled input material. Target gene expression was then normalized to β-actin expression and expression in siScrambled-treated cells was set to 1. Sequences of qPCR primers are listed in Table S2.

### Genome-editing using CRISPR/Cas9

HeLa cells were transfected with fluorescently labelled mKate2-Cas9 mRNA (Horizon), tracrRNA (Horizon) and crRNA (5’-TTTGTTTGCGGGTCACTTCA, Horizon) using Dharmafect DUO (Horizon) according to the manufacturer’s instructions. The following day, single mKate2-positive cells were sorted into 96-wells using fluorescence-activated cell sorting and single-cell clones were expanded. For screening of clones, genomic DNA was extracted using DirectPCR Lysis Reagent (Viagen Biotech) containing 1 mg/mL proteinase K (Sigma-Aldrich) and the targeted genomic region was amplified by PCR (fwd: 5′-CCATTGCCACCATC CATTCTGC, rev.: 5’-GGAAGCTTCAACAGACTTGGACC) followed by Sanger-sequencing. Sequencing results were analysed using the ICE analysis tool v2 (Synthego, https://ice.synthego.com/) to infer the identity and contribution of individual alleles in each clone.

### Generation of mouse embryonic fibroblasts

Primary mouse embryonic fibroblasts (MEFs) were obtained from E13.5 embryos from timed pregnancies with the day of the copulation plug counted as E0.5. Decapitated embryos were minced and cells dissociated using trypsin. Single cell suspensions were allowed to adhere to culture dishes in DMEM with 4.5 g/L glucose, 10% fetal bovine serum and 100 U/mL penicillin/streptomycin at 37°C and 5% CO_2_. Early passage primary MEFs (P2 to P5) were immortalized by retroviral transduction with a p53-shRNA construct and selected with 2 μg/mL puromycin for 10-14 days.

### Surface biotinylation β1-integrin endocytosis

SiRNA-treated HeLa cells were starved in serum-free medium for 45 min, transferred to ice and washed twice with PBS. Surface-exposed proteins were biotinylated using 0.5 mg/mL Sulfo-NHS-SS-Biotin (ThermoFisher Scientific) in PBS at 4°C. Cells were washed twice with 50 mM glycine in TBS to stop the biotinylation reaction. For integrin internalization, cells were incubated with pre-warmed full medium for 10 – 30 min at 37°C. Surface-exposed biotin was cleaved using 50 mM TCEP (Carl Roth) in TBS. Cells were lysed in Biotin-LB (75 mM Tris-HCl pH 7.5, 200 mM NaCl, 7.5 mM EDTA, 7.5 mM EGTA, 1.5% Triton X-100, 0.75% Igepal CA-630). Biotinylated proteins were purified using magnetic streptavidin beads (Pierce, ThermoFisher Scientific). Lysates and purified biotinylated proteins were analysed by immunoblotting using detection by fluorescent antibody-conjugates (LI-COR Biosciences).

### Measurement of mitotic rounding

siRNA-treated HeLa cells were seeded to matrigel-coated (Sigma-Aldrich), glass-bottomed plates (Mattek) 24 hours before imaging. Wide field, brightfield time-lapse imaging at 37°C was carried out on a Nikon Ti inverted microscope at 5 minute intervals using a 20×(Plan Fluor ELWD Ph1 NA 0.45, WD 7.4) objective. Images were processed and analysed using ImageJ (Schindelin et al., 2012). For cell rounding rate analysis, cells were aligned so that time-point 0 represents nuclear envelope breakdown (NEB). Cell outlines for 20 cells in each condition were manually segmented from consecutive bright-field images from 30 minutes before NEB until the end of metaphase (defined as the frame before anaphase) using the polygon selections tool in ImageJ. Cell area was measured at every time-point and rounding rate was calculated as the difference in cell area between consecutive time-points divided by the time difference.

## Quantification and Statistical Analysis

### Image analysis

All image analysis was performed using ImageJ (Schindelin et al., 2012). For analysis of live imaging data, time lapse series were corrected for xy-shifts in between frames using the ImageJ plug-in “Correct 3D drift” (Parslow et al., 2014). For analysis of paxillin-labelled adhesion lifetimes, we used the plug-in “TrackMate” (Tinevez et al., 2017), with following parameters: estimated blob diameter 2 μm; threshold 0.3; individual filtering by quality to match bona fide adhesions; LAP tracker: max. frame to frame distance 2 μm, gap max. distance 2 μm, max. frame gap 1.5 μm, splitting and merging up to 1.5 μm; excluded tracks with a lifetime of less than 30 min. For experiments with addition of para-Nitroblebbistatin, background subtraction was used in the 488 channel from a ROI drawn outside of the cell to compensate for the increase in background fluorescence caused by the fluorescent properties of para-Nitroblebbistatin. For quantification of protein recruitment to adhesions as labelled by paxillin, we identified the ratio of mean intensity at adhesions over the mean intensity in the rest of the cell as the most robust read-out. This allows for unbiased quantification without the need of subjective intensity thresholding and eliminates increases in fluorescence in the cell caused by blebbistatin addition that had not been removed by background subtraction.

### Statistics

Statistical analyses were performed with Graphpad Prism 9 software and details of all statistical analyses are provided in the respective figure legends. For all tests, the levels of statistical significance were defined as: *P < 0.05, **P < 0.01, ***P < 0.001 and ****P < 0.0001. When two experimental conditions or groups were being compared, we used an unpaired two-tailed t-test with Welch’s correction to compensate for the possibility of unequal variances in the two conditions. For comparisons between more than two experimental conditions, we used analysis of variance (ANOVA) followed by Tukey’s test (for comparisons between all conditions and groups) or Šídák’s test (for multiple pairwise comparisons) to compute *P* values. *P* values from ANOVAs are reported as multiplicity-adjusted *P* values.

## Supplementary Material

Article & Supplementary information

Supplementary information; Figures S1-S7 & Tables S1-S3

Video S1

Video S2

Video S3

Video S4

## Data Availability

All original data reported in this paper will be shared by the lead contact upon request. This paper does not report original code. Any additional information required to reanalyze the data reported in this paper is available from the lead contact upon request.

## In brief

Remodeling of cell-matrix adhesions is crucial for development and tissue homeostasis. Posor, Kampyli et al. show that synthesis of the phosphatidylinositol-3,4-bisphosphate (PtdIns(3,4)P_2_) membrane lipid promotes disassembly of focal adhesions by inactivating the RhoA small GTPase. This identifies a pathway that links cessation of growth factor signaling to adhesion disassembly through a membrane lipid switch.

## Highlights

- PtdIns(3,4)P_2_ produced by PI3K-C2β promote focal adhesion disassembly
- PI3K-C2β recruitment to adhesions is regulated via phosphorylation by PKN2
- Local PtdIns(3,4)P_2_ formation inactivates RhoA through recruitment of the ARAP3 GAP
