# Supplementary material for: Local synthesis of the phosphatidylinositol-3,4-bisphosphate lipid drives focal adhesion turnover
Source: Dev Cell. 2022 Jul 25;57(14):1694–1711.e7. doi: 10.1016/j.devcel.2022.06.011 (PMC7613278; doi:10.1016/j.devcel.2022.06.011)
Supplement: Document S1. Figures S1–S7 and Tables S1–S3 [file mmc1.pdf]

Developmental Cell, Volume *57*

Supplemental information

Local synthesis of the phosphatidylinositol-3,4-bisphosphate lipid drives focal adhesion turnover

York Posor, Charis Kampyli, Benoit Bilanges, Sushila Ganguli, Philipp A. Koch, Alexander Wallroth, Daniele Morelli, Michalina Jenkins, Samira Alliouachene, Elitza Deltcheva, Buzz Baum, Volker Haucke, and Bart Vanhaesebroeck

Figure S1

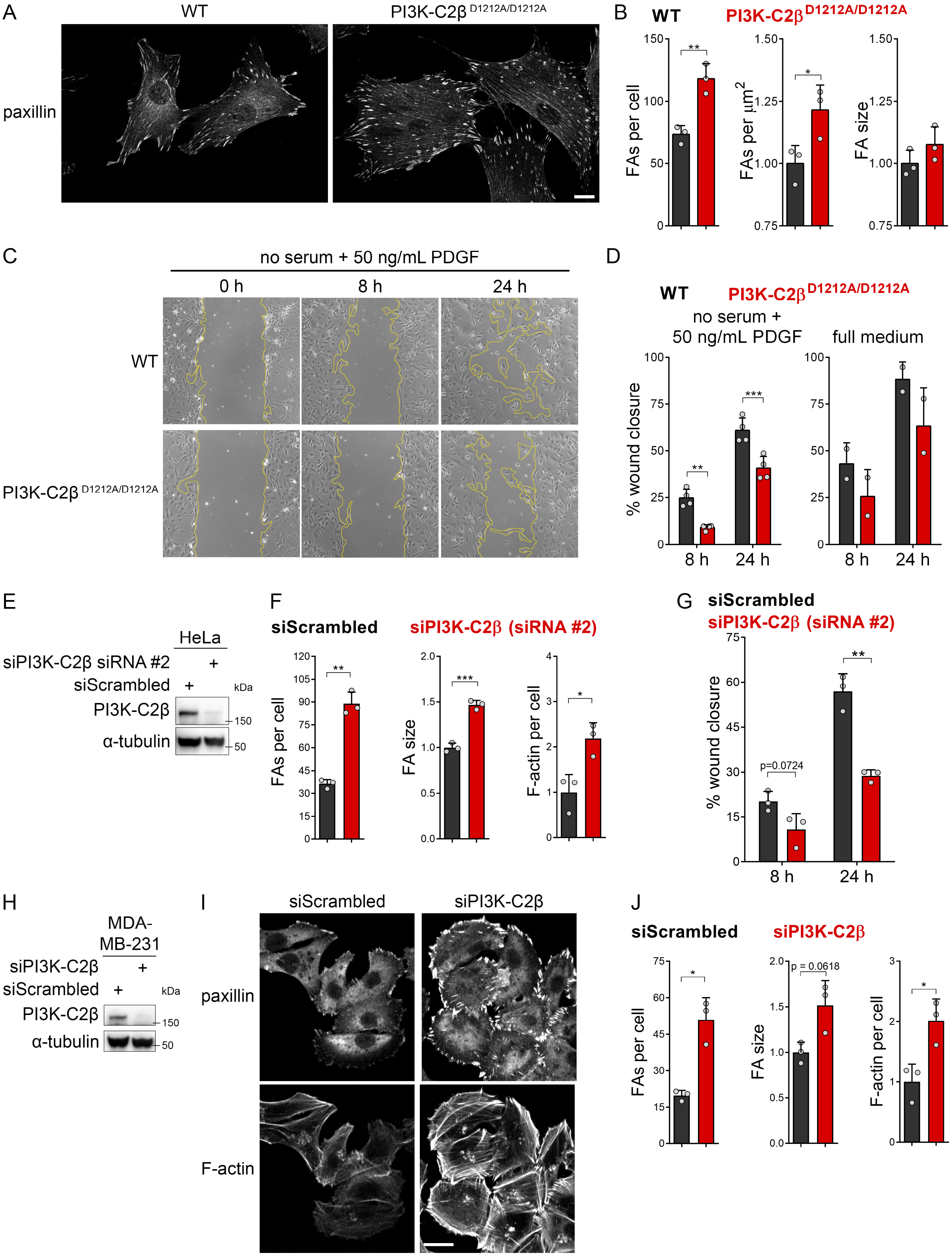
A
WT
PI3K-C2β D1212A/D1212A
paxillin
B
WT
PI3K-C2β D1212A/D1212A
FAs per cell
FAs per µm2
FA size
C
no serum + 50 ng/mL PDGF
0 h
8 h
24 h
WT
PI3K-C2β D1212A/D1212A
D
WT
PI3K-C2β D1212A/D1212A
no serum + 50 ng/mL PDGF
full medium
% wound closure
8 h
24 h
E
HeLa
siPI3K-C2β siRNA #2
siScrambled
kDa
PI3K-C2β
α-tubulin
150
50
F
siScrambled
siPI3K-C2β (siRNA #2)
FAs per cell
FA size
F-actin per cell
G
siScrambled
siPI3K-C2β (siRNA #2)
% wound closure
p=0.0724
8 h
24 h
H
MDA-MB-231
siPI3K-C2β
siScrambled
kDa
PI3K-C2β
α-tubulin
150
50
I
siScrambled
siPI3K-C2β
paxillin
F-actin
J
siScrambled
siPI3K-C2β
FAs per cell
FA size
F-actin per cell
p = 0.0618

Figure S1: Accumulation of focal adhesions and impaired migration in fibroblasts from mice with kinase-inactive PI3K-C2β, and HeLa or MDA-MB-231 cells depleted of PI3K-C2β.

(A-D) Mouse embryonic fibroblasts (MEFs) were obtained from *Pik3c2b*$^{wt/wt}$ or *Pik3c2b*$^{D1212A/D1212A}$ mice and immortalized by stable p53-knockdown. The D1212A mutation abolishes kinase activity.

(A, B) Accumulation of focal adhesions in MEFs from PI3K-C2β-kinase-inactive mice. MEFs were stained for paxillin and imaged by confocal laser scanning microscopy. (A) Bar, 20 µm. (B) Mean + s.d. from n = 3 independent experiments; unpaired two-tailed t-test with Welch's correction.

(C, D) Impaired migration in MEFs from PI3K-C2β-kinase-inactive mice in a scratch wound assay in presence of only PDGF but no serum (C and D) or complete medium with 10 % serum (D). Wound closure was assessed after 8 h and 24 h. (C) Bar, 200 µm. (D) Mean + s.d. from n = 4 independent experiments, two-way ANOVA with Sidak's test.

(E, F, G) Accumulation of focal adhesions, F-actin and impaired migration in HeLa cells depleted of PI3K-C2β using an alternative siRNA, siRNA #2. HeLa cells were depleted of PI3K-C2β by siRNA transfection. (E) Immunoblots of cell extracts representative of at least 3 independent experiments. Tubulin was detected as loading control. (F) Cells were stained for paxillin and for F-actin using phalloidin, followed by imaging by spinning disk confocal microscopy. Mean + s.d. from n = 3 independent experiments, unpaired two-tailed t-test with Welch's correction. (G) Scratch wound assay, with wound closure assessed after 8 h and after 24 h in complete medium containing serum and mitomycin C to block proliferation. Mean + s.d. from n = 3 independent experiments, unpaired two-tailed t-test with Welch's correction.

(H, I, J) Accumulation of focal adhesions and F-actin in cells of the migratory breast cancer line MDA-MB-231 depleted of PI3K-C2β by siRNA transfection. (H) Immunoblots of cell extracts representative of 3 independent experiments. Tubulin was detected as loading control. (I, J) Cells were stained for paxillin and for F-actin using phalloidin, followed by imaging by spinning disk confocal microscopy. (I) Bar, 20 µm. (J) Mean + s.d. from n = 3 independent experiments, unpaired two-tailed t-test with Welch's correction.

Related to Fig. 1. FAs, focal adhesions.

Figure S2

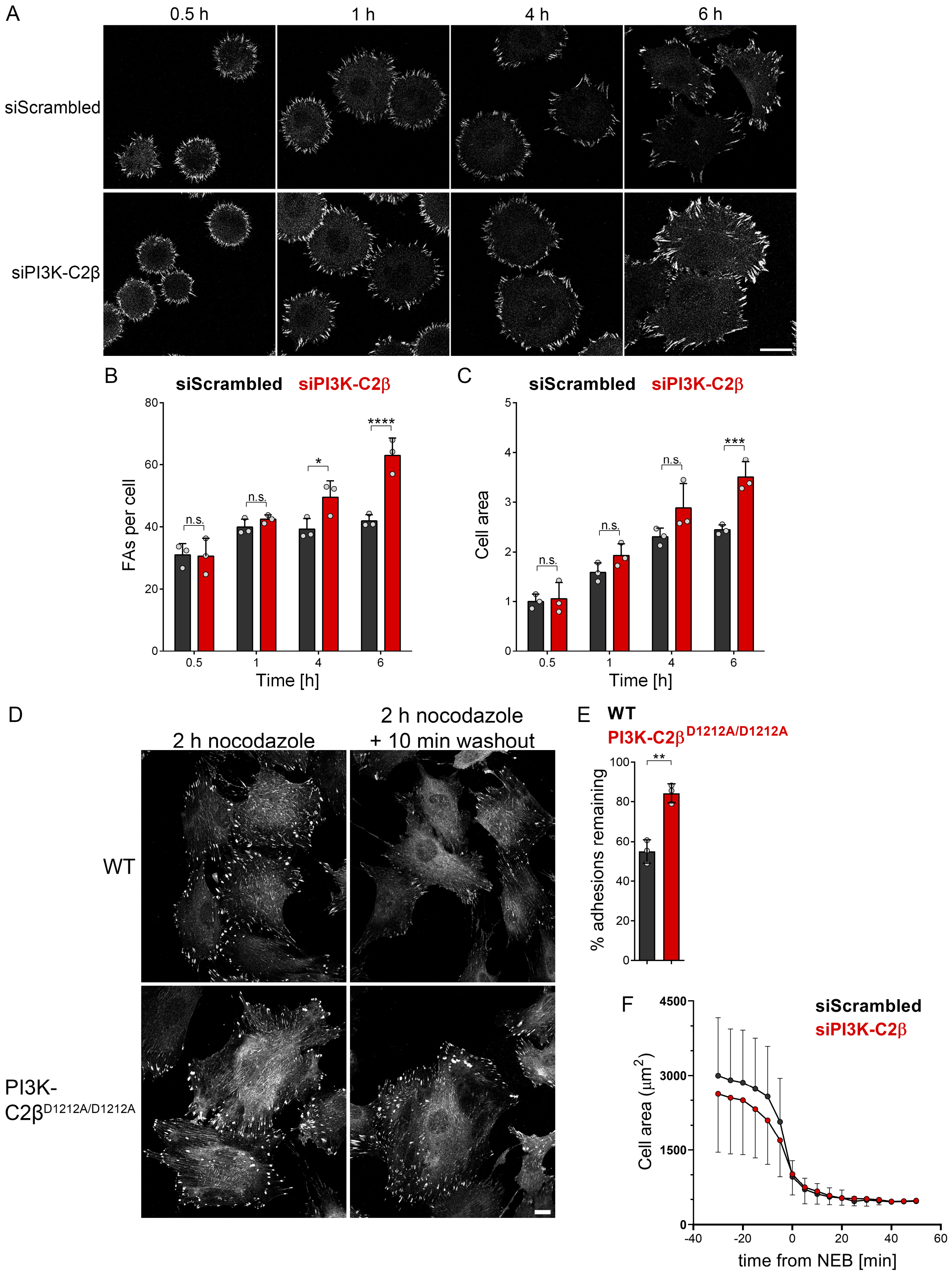

A
0.5 h
1 h
4 h
6 h
siScrambled
siPI3K-C2β
B
siScrambled
siPI3K-C2β
FAs per cell
n.s.
n.s.
*
****
Time [h]
C
siScrambled
siPI3K-C2β
Cell area
n.s.
n.s.
n.s.
***
Time [h]
D
2 h nocodazole
2 h nocodazole
+ 10 min washout
WT
PI3K-C2β$^{D1212A/D1212A}$
E
WT
PI3K-C2β$^{D1212A/D1212A}$
% adhesions remaining
**
F
siScrambled
siPI3K-C2β
Cell area (μm^2)
time from NEB [min]

Figure S2: Perturbation of PI3K-C2β impairs focal adhesion disassembly but not *de novo* adhesion formation.

(A - C) HeLa cells depleted of PI3K-C2β were trypsinized to cleave surface adhesion molecules and re-seeded on Matrigel-coated coverslips. Cells were allowed to adhere and spread for 0.5 to 6 h before staining for paxillin and imaging by laser scanning confocal microscopy (A) Bar, 20 µm. (B) The number of focal adhesions in PI3K-C2β-depleted cells only starts to diverge from control cells at 4 h after plating. Mean + s.d. from n = 3 independent experiments, two-way ANOVA with Sidak's test. (C) Cell area in PI3K-C2β-depleted cells only begins to differ from control cells at 4-6 h after plating. Mean + s.d. from n = 3 independent experiments, two-way ANOVA with Sidak's test.

(D, E) Cell-matrix adhesion disassembly is impaired in MEFs from PI3K-C2β-kinase-inactive mice. Adhesion disassembly was stalled for 2 h by nocodazole treatment. Synchronous adhesion disassembly was then triggered by nocodazole washout. After 10 min, cells were fixed, stained for paxillin and imaged by laser scanning confocal microscopy. (D) Bar 20 µm. (E) Adhesions remaining 10 min after nocodazole washout (in percent of adhesions at 2 h nocodazole treatment). Mean + s.d. from MEF cell lines obtained from n = 3 mouse embryos for WT and PI3K-C2β$^{D1212A/D1212A}$ each, with data for each cell line obtained by averaging over two to three independent experiments; unpaired two-tailed t-test with Welch's correction.

(F) The rate of cell area change during mitotic rounding is not significantly affected in HeLa cells depleted of PI3K-C2β. HeLa cells were imaged by phase-contrast time-lapse microscopy and the cell area was measured at time points before and after nuclear envelope breakdown (NEB). Mean + or – s.d. from n = 20 cells per condition.

Related to Fig. 2. FAs, focal adhesions.

Figure S3

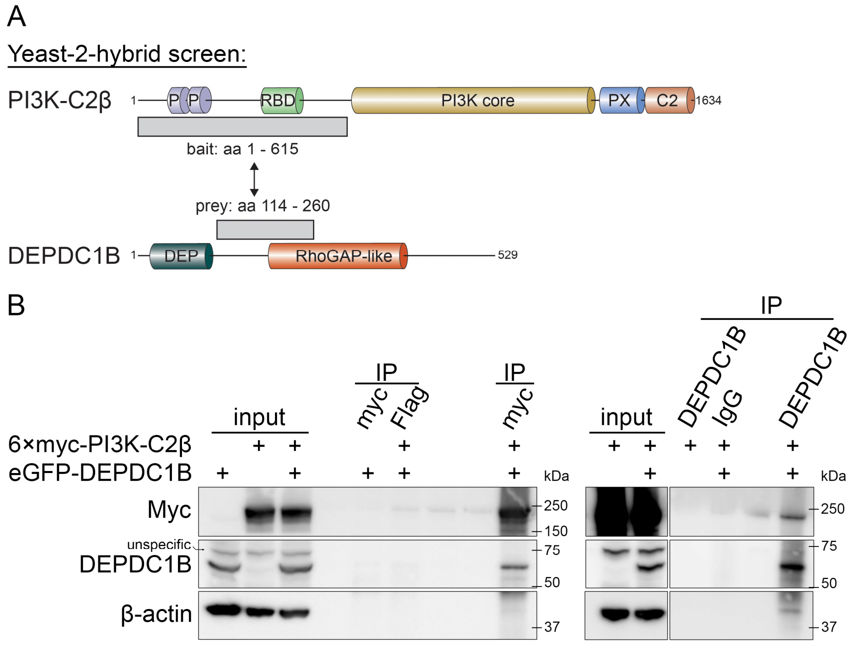

Figure S3: Identification of DEPDC1B as an interaction partner of PI3K-C2β.

(A) Scheme depicting a yeast-2-hybrid screen using the N-terminal 615 amino acids of PI3K-C2β as a bait. This screen identified DEPDC1B, a protein previously implicated in focal adhesion disassembly, as an interaction partner of PI3K-C2β. The fragment of DEPDC1B that interacted with the bait encompasses amino acids 114 – 260 of human DEPDC1B.

(B) Validation of DEPDC1B as an interaction partner of PI3K-C2β. HEK293 cells transiently expressing 6×myc-PI3K-C2β, eGFP-DEPDC1B or both were subjected to immunoprecipitation of myc or eGFP. Immunoprecipitation of either 6×myc-PI3K-C2β or eGFP-DEPDC1B co-precipitated the other protein, but not β-actin. For the right panel, irrelevant lanes in between input and IPs have been spliced out but all lanes originate from the same blot. Data shown are representative of at least 5 (from 6×myc-PI3K-C2β side) or 2 (from eGFP-DEPDC1B side) independent experiments.

Related to Fig. 4.

Figure S4

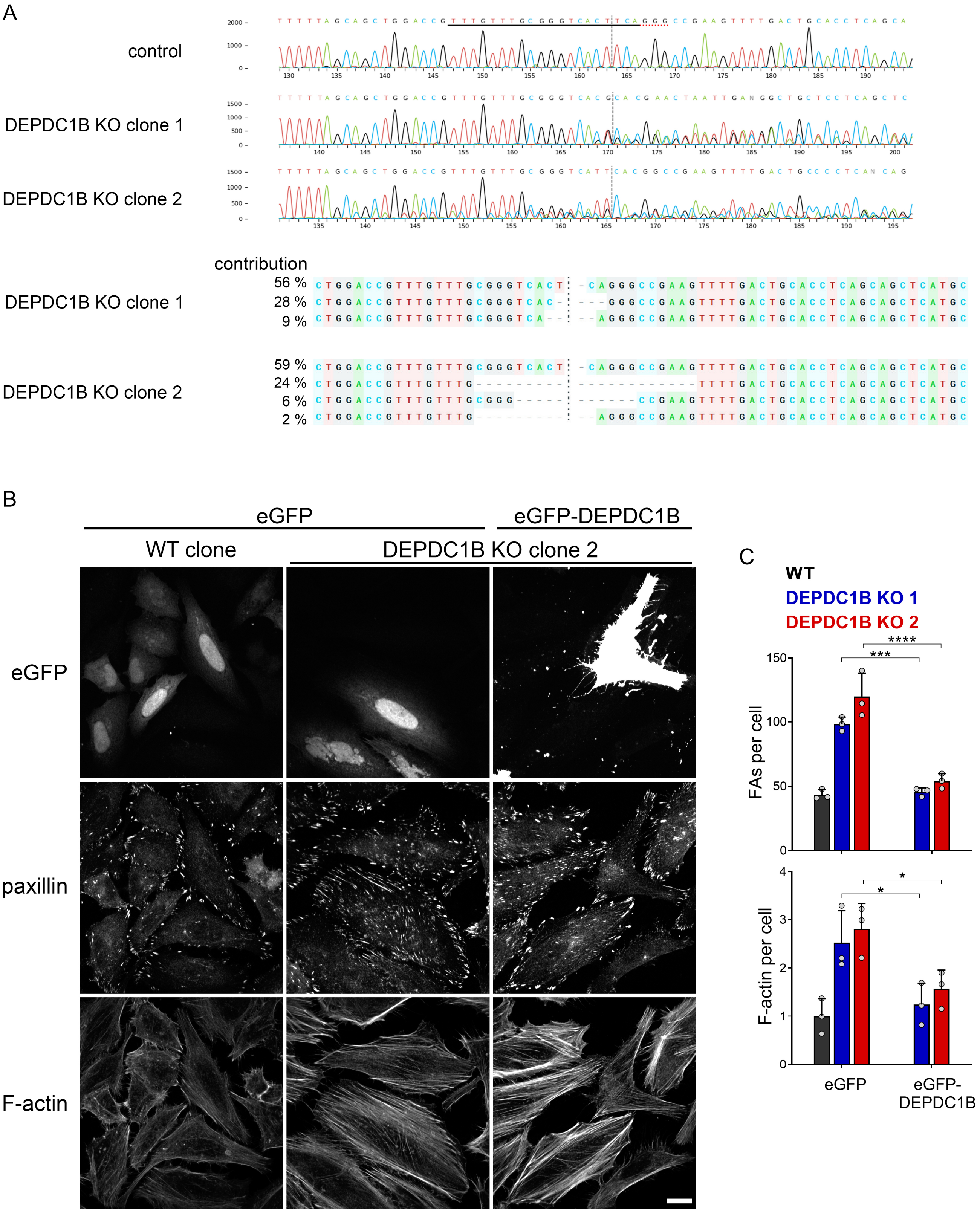
A
control
DEPDC1B KO clone 1
DEPDC1B KO clone 2
contribution
56 %
28 %
9 %
59 %
24 %
6 %
2 %
B
eGFP
eGFP-DEPDC1B
WT clone
DEPDC1B KO clone 2
eGFP
paxillin
F-actin
C
WT
DEPDC1B KO 1
DEPDC1B KO 2
FAs per cell
F-actin per cell
eGFP
eGFP-DEPDC1B
****
***
*
*

Figure S4: Knockout of DEPDC1B in HeLa cells phenocopies depletion of PI3K-C2β.

(A) HeLa cells were genome-targeted to generate DEPDC1B knockout (KO) cells using CRISPR/Cas9. Single cell clones were expanded and subjected to Sanger sequencing, followed by analysis of the contribution of individual alleles using the ICE tool (https://ice.synthego.com/#/). The upper panel shows sequencing traces for two clones DEPDC1B KO 1 and KO 2 in comparison to a non-edited sample. The sequence of the crRNA (the targeting sequence within the guide RNA) used is underlined and the designated cut site is indicated with a dashed line. The lower panel shows the inferred contribution of individual alleles. Both clones showed >90% predicted KO efficiency.

(B, C) KO of DEPDC1B phenocopies depletion of PI3K-C2β. HeLa clones either WT or KO for DEPDC1B were transiently transfected with eGFP or eGFP-DEPDC1B. Cells were stained for paxillin and for F-actin using phalloidin and imaged by spinning disk confocal microscopy. (B) Bar 20 μm. (C) Mean + s.d. from n = 3 independent experiments, two-way ANOVA with Sidak's test.

Related to Fig. 4. FAs, focal adhesions.

Figure S5

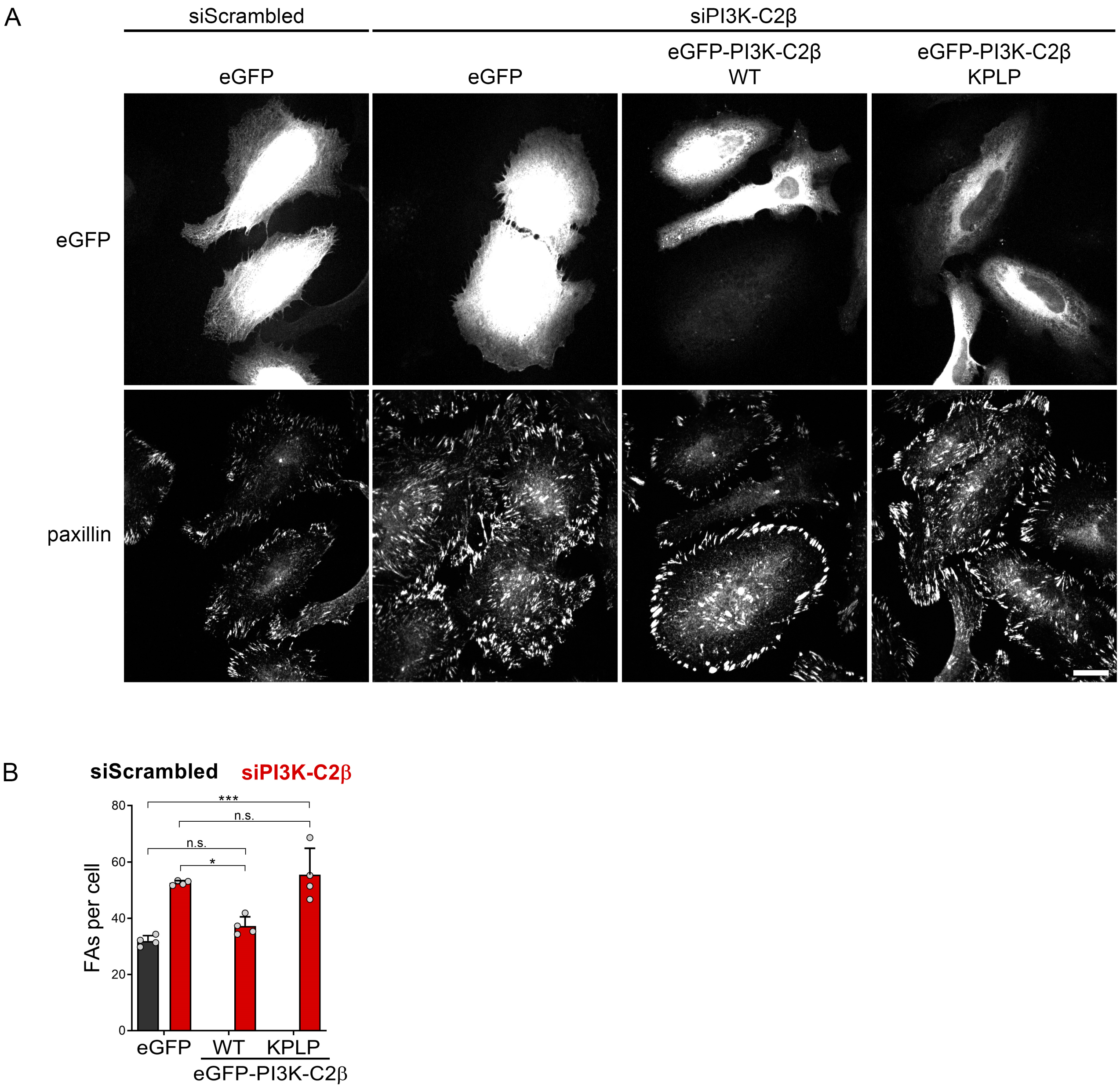

Figure S5: A mutant PI3K-C2β which can only produce PtdIns(3)P does not rescue accumulation of focal adhesions in PI3K-C2β-depleted cells.

(A, B) Mutation of residues ^{1228}KRDR^{1231} to ^{1228}KPLP^{1231} in the substrate-binding activation loop renders PI3K-C2β incapable of producing PtdIns(3,4)P_2, whereas synthesis of PtdIns(3)P remains unperturbed. HeLa cells depleted of PI3K-C2β using siRNAs were transfected with eGFP or either siRNA-resistant WT or KPLP-mutant eGFP-PI3K-C2β and stained for paxillin. Images were acquired using a spinning disk confocal microscope. (A) Bar 20 µm. (B) Mean + s.d. from n = 4 independent experiments, 2-way ANOVA with Tukey's test.

Related to Fig. 5.

Figure S6

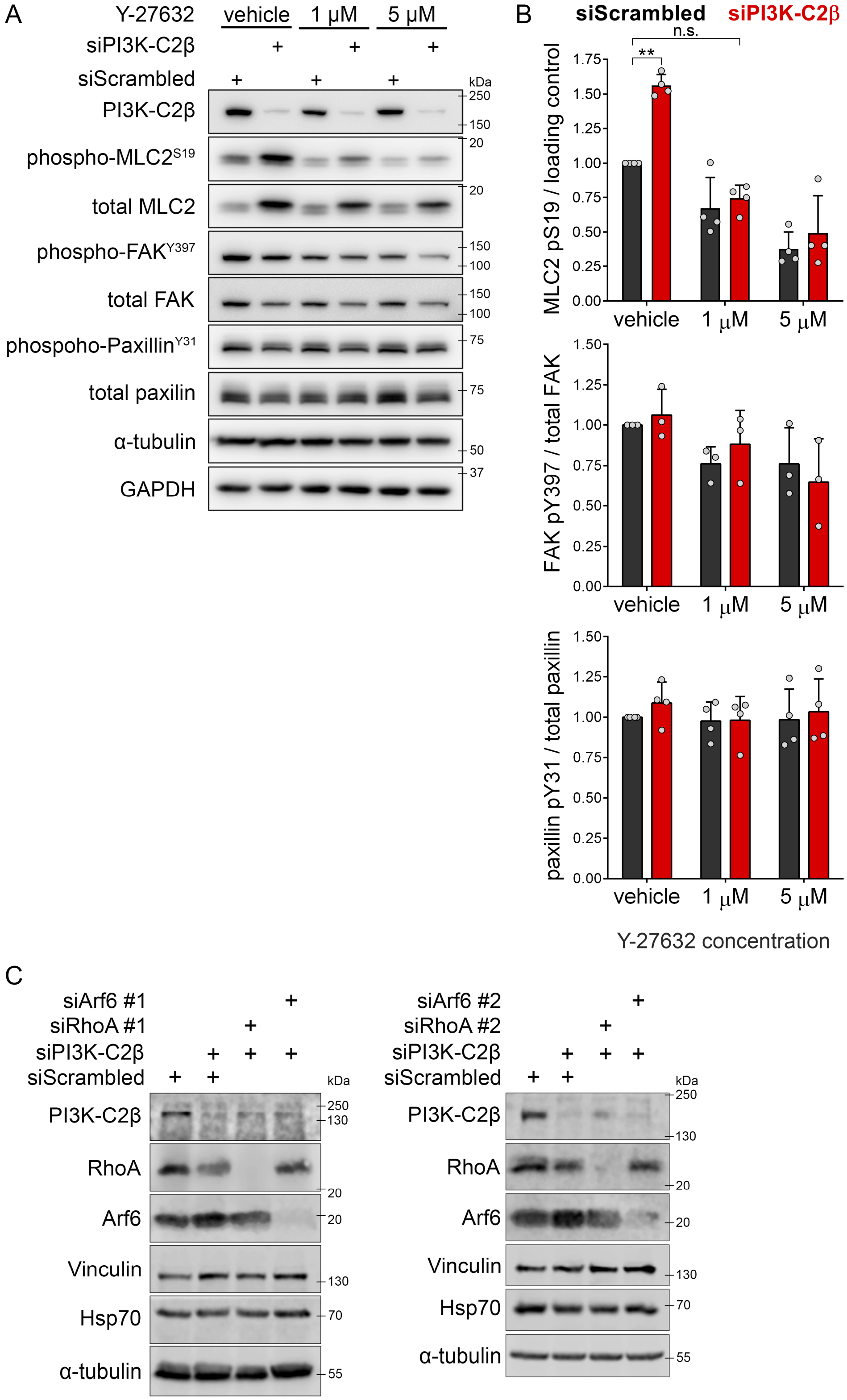

Figure S6: ROCK inhibition rescues increased phospho-MLC2^{S19} levels in PI3K-C2β-depleted cells.

(A, B) HeLa cells depleted of PI3K-C2β using siRNAs were treated for 16 h (overnight) with the ROCK inhibitor Y-27632 and cell lysates analysed by immunoblotting for the indicated proteins. A low dose of Y-

27632 (1 μM) reduces phospho-MLC2^{S19} in PI3K-C2β-depleted cells to control levels. (A) Data shown are representative of four independent experiments. (B) Densitometric analysis of immunoblots as shown in (A). Mean + s.d. from n = 4 (MLC2 pS19, paxillin pY31) or n = 3 (FAK pY397) independent experiments, two-way ANOVA with Tukey's test.

(C) Co-depletion of PI3K-C2β with RhoA or Arf6 using siRNAs. Representative blots from two independent experiments are shown. Vinculin, Hsp70 and α-tubulin were detected as loading controls.

Related to Fig. 6.

Figure S7

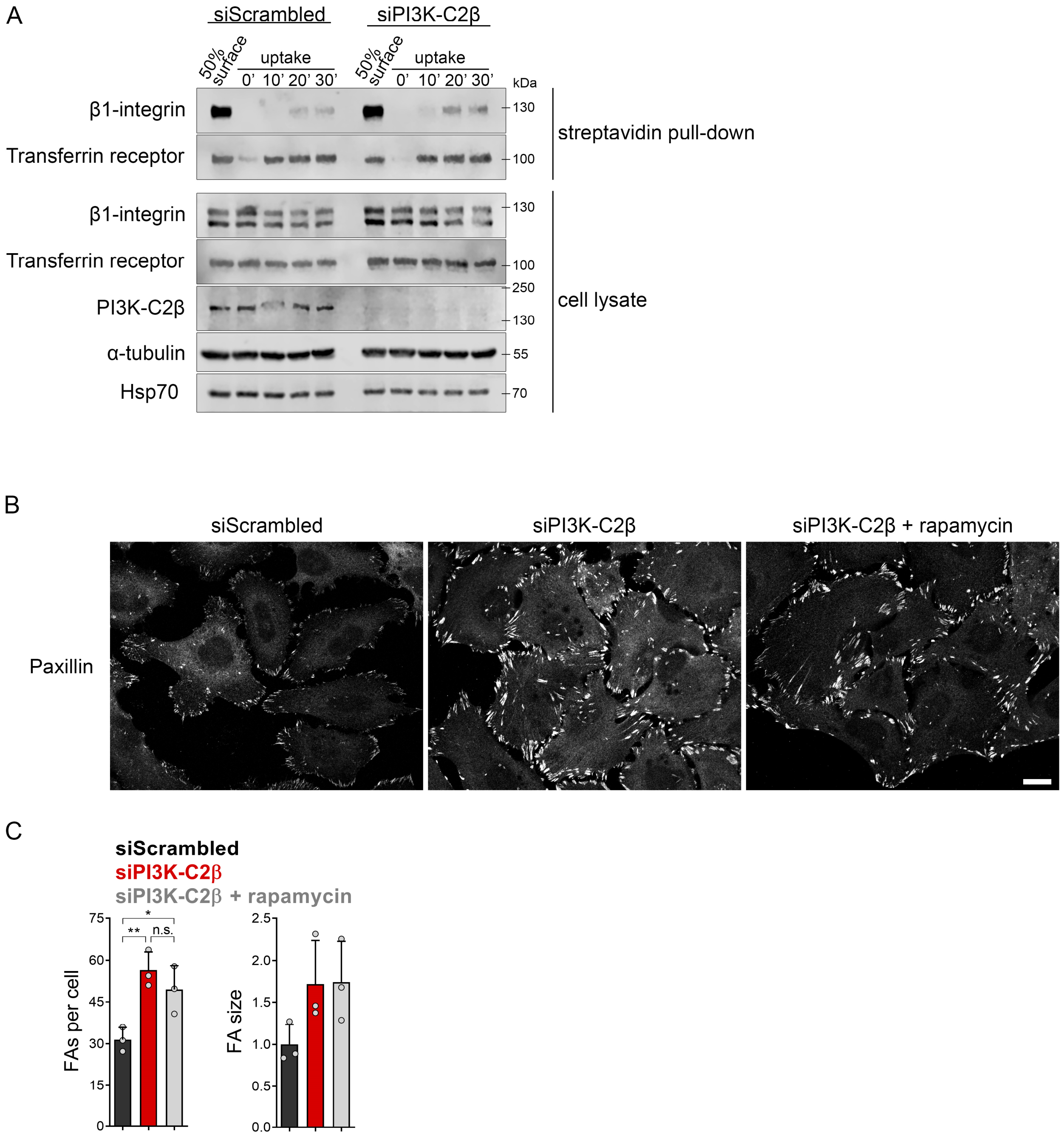

Figure S7: Total β1-integrin endocytosis is unaffected and Rapamycin does not rescue accumulation of focal adhesions in cells depleted of PI3K-C2β.

(A) HeLa cells were depleted of PI3K-C2β using siRNAs and the amount of β1-integrin internalized after different time points was measured using a surface-biotinylation and streptavidin-pull-down assay (see STAR methods). In brief, after incubation at 37°C for the indicated time (uptake), surface-exposed biotin was cleaved using TCEP and biotinylated proteins were purified using magnetic streptavidin beads. Levels of proteins in lysates or purified samples were analysed using fluorescent detection of immunoblots. The blots shown are representative of four independent experiments.

(B, C) HeLa cells depleted of PI3K-C2β were treated overnight with vehicle or 100 nM rapamycin, stained for paxillin and imaged by confocal microscopy. (A) bar 20 µm. (B) Mean + s.d., ordinary one-way ANOVA with Tukey's test. FAs, focal adhesions.

Related to Fig. 6 and 7.

Supplementary table 1

Supplementary table 1: Antibodies used in this study. Related to STAR Methods.

antigen	host species	IF dilution	blotting dilution	manufacturer	order number
Paxillin	mouse	1:400	1:4000	BD Bioscience	610051
phospho-MLC2 S19	mouse	1:100	1:1000	Cell Signaling Technology	3675
total MLC2	rabbit		1:1000	Cell Signaling Technology	3672
α-tubulin (clone B-5-1-2)	mouse	1:400	1:4000	Sigma	T5168
c-myc (clone 9E10)	mouse		1:1000	Abcam	ab32
FLAG	mouse			Merck (Sigma-Aldrich)	F-3165
Mouse IgG	rabbit			Merck (Sigma-Aldrich)	M-7023
DEPDC1B	rabbit			Biorbyt	orb183064
DEPDC1B	rabbit		1:500	MyBiosource	mbs154376
DEPDC1B (clone 2H2)	mouse		1:500	Merck (Sigma-Aldrich)	SAB1403301
β-actin (clone AC15)	mouse		1:10,000	Merck (Sigma-Aldrich)	A1978
PI3K-C2β	mouse		1:250	BD Bioscience	611342
Talin	mouse		1:500	Merck (Sigma-Aldrich)	T3287
eGFP	rabbit	1:800	1:20,000	Abcam	ab6556
Erk1/2	rabbit		1:1,000	Cell Signaling Technology	9102
pan 14-3-3	mouse		1:500	Santa Cruy Biotechnology	sc-1657
RhoA	rabbit		1:250	Cell Signaling Technology	2117
Rac1 (clone 23A8)	mouse		1:1,000	Merck	05-389
Cdc42	rabbit		1:500	Cell Signaling Technology	2462
vinculin	mouse		1:10,000	Merck (Sigma-Aldrich)	V9131
R-Ras	rabbit		1:250	Cell Signaling Technology	8446
H/K/N-Ras	mouse		1:250	BD Bioscience	610001
ARAP3	rabbit		1:500	Novus	NBP1-84541
GAPDH	mouse		1:10,000	Abcam	ab8245
phospho-FAK Y397	rabbit		1:500	Cell Signaling Technology	3283
total FAK	mouse		1:500	BD Bioscience	610087
phospho-paxillin Y31	rabbit		1:3,000	Abcam	ab32115
Arf6 (C-term.)	rabbit		1:200	Gift from J. Donaldson Song et al., J Cell Sci (1998) 111 (15): 2257–2267.	-
Arf1	mouse		1:500	Abcam	ab18108
Hsp70	mouse		1:5,000	Invitrogen (ThermoFischer)	MA3-006
β1-integrin	mouse		1:400	BD Bioscience	610467
Transferrin receptor	rabbit		1:1,000	Merck (Sigma-Aldrich)	HPA028598

Supplementary table 2

Supplementary table 2: siRNAs and qPCR primers used. Related to STAR Methods.

**siRNA sequences**				
**target (all human)**	**number if pool-of-four**	**sequence (5'-3')**	**reference**	**manufacturer**
non-targeting (siScrambled)		AUUGUUAACCGUAUUCUUA		Eurofins
PI3K-C2β #1		GCUACCAGCUAUGAAGAUU	Marat et al. Science **356,** 2017 ("siRNA #2")	Eurofins
PI3K-C2β #2		GUUCGACACUUACCACAAU	Marat et al. Science **356,** 2017 ("siRNA #1")	Eurofins
DEPDC1B	#1	GUACAAGCGUCACAGUAUU		Horizon Discovery # L-013830-00-0005
	#2	CGAAGUUCAUCAUCCAUAA		
	#3	GUACUGGGUUUGUUACAGA		
	#4	GCGUGUGGCUCAUCUACGA		
ARAP3	#1	GUAAUGAGAUAGUACAGUU	Gambardella et al., Sci Signal **3**, 2010	Eurofins
	#2	GCAGAAAUGUGCGGCUCUA		Eurofins
	#3	ACACGGGAGUGGACAGUGA		Eurofins
	#4	GAACGGGAGUGGCCUUUGG		Eurofins
RhoA #1		GAUACCGAUGUUAUACUGAUGUGUU	Marchesi et al., Dev Cell 31, 2014	Sigma-Aldrich
RhoA #2	#1	CGACAGCCCUGAUAGUUUA		Horizon Discovery # L- 003860-00-0005
	#2	GACCAAAGAUGGAGUGAGA		
	#3	GCAGAGAUAUGGCAAACAG		
	#4	GGAAUGAUGAGCACACAAG		
Arf6 #1	#1	CGGCAUUACUACACUGGGA		Horizon Discovery # L-004008-00-0005
	#2	UCACAUGGUUAACCUCUAA		
	#3	GAGCUGCACCGCAUUAUCA		
	#4	GAUGAGGGACGCCAUAAUC		
Arf6 #2		AACCCAUUCAUAGGAUUAU	Moravec et al., J Biol Chem **287**, 2012 ("Arf6–1")	Eurofins

**qPCR primers**		
**target gene / primer**	**amplificate length**	**sequence (5'-3')**
DEPDC1B fw	182 bp	GAGCTACCAGGCTGTGGAATG
DEDC1B rev	182 bp	CGTTTGTTTGCGGGTCACTT

Supplementary table 3

Supplementary table 3: Inhibitors and small molecules used in this study. Related to STAR Methods.

Compound	concentration used	Supplier	order number
Phalloidin-AlexaFluor647	1:500	ThermoFisher Scientific	A22287
Nocodazole	10 µM	Merck (Sigma-Aldrich)	M1404
para-Nitroblebbistatin	25 µM	Axol	ax494693
PKC412	1 µM	Selleck Chemicals	S8064
Cdk1/2 inhibitor III	2 µM	EMD Millipore	S217714
GDC-0941	250 nM	ApexBio via Stratech	A3432-APE
PI-103	1 µM	EMD Millipore	528100
Y-27632	1 µM	Cayman Chemical	10005583
Rapamycin	100 nM	Calbiochem	553210
Mitomycin C	1 µg/mL (HeLa), 10 µg/mL (MEFs)	Merck (Sigma-Aldrich)	M4287
GTPγS	0.2 mM	Merck (Sigma-Aldrich)	G8634
